# Deficiency of 2‐Oxoglutarate Carrier (Slc25a11) Drives RPE Epithelial‐to‐Mesenchymal Transition and Exacerbates Subretinal Fibrosis in Neovascular Age‐Related Macular Degeneration

**DOI:** 10.1111/acel.70271

**Published:** 2025-10-28

**Authors:** Mo Wang, Feng‐Juan Gao, Wenyi Tang, Boya Lei, Fangyuan Hu, Jun Ma, Srinivas R. Sadda, Ram Kannan, Parameswaran G. Sreekumar, Gezhi Xu

**Affiliations:** ^1^ Shanghai Eye Diseases Prevention &Treatment Center/Shanghai Eye Hospital, School of Medicine Tongji University Shanghai China; ^2^ Eye Institute, Eye and ENT Hospital, College of Medicine Fudan University Shanghai China; ^3^ Shanghai Key Laboratory of Visual Impairment and Restoration, Science and Technology Commission of Shanghai Municipality Shanghai China; ^4^ Jules Stein Eye Institute, David Geffen School of Medicine University of California at Los Angeles Los Angeles California USA; ^5^ Department of Ophthalmology, David Geffen School of Medicine University of California‐Los Angeles Los Angeles California USA; ^6^ Doheny Eye Institute Pasadena California USA

**Keywords:** mitochondrial dysfunction, neovascular AMD, OGC, RPE‐EMT, subretinal fibrosis

## Abstract

Subretinal fibrosis significantly contributes to vision loss in neovascular age‐related macular degeneration (nAMD). Epithelial‐to‐mesenchymal transition (EMT) in RPE cells is a key early step in subretinal fibrosis. While mitochondrial dysfunction in RPE is involved, the metabolic and molecular connections between EMT and mitochondria are not well understood. This study explores the role of oxoglutarate carrier (OGC; Slc25a11) on EMT and investigates the molecular mechanisms, focusing on its role in mitochondrial metabolism and GSH transport. OGC‐silenced or overexpressed ARPE‐19 cells were treated with TGF‐β2 (10 ng/mL) for 48 h. EMT markers, cell migration, mtGSH, and mitochondrial bioenergetics and signaling pathways were assessed. In vivo, subretinal fibrosis was induced in wild‐type and OGC^+/−^ mice via laser photocoagulation. Fibrosis volume was measured using optical coherence tomography and immunostaining in RPE‐choroid flat mounts. OGC silencing aggravated EMT, while overexpression attenuated TGF‐β2‐induced EMT, cell proliferation, and migration. OGC knockdown significantly enhanced RPE EMT, as evidenced by upregulated expression of α‐SMA, fibronectin, collagen type I, and Slug, while E‐cadherin was downregulated. OGC overexpression improved mitochondrial bioenergetics, whereas its inhibition reduced mitochondrial respiration, which was further aggravated by co‐treatment with TGF‐β2. Loss of OGC promoted cell proliferation and migration through Slug‐mediated EMT. OGC depletion stimulated EMT via pSmad2/3 upregulation, dependent on the PI3K/AKT signaling pathway activation. In vivo studies further demonstrate that subretinal fibrosis was significantly augmented in OGC^+/−^ mice via TGF‐β2‐dependent PI3K signaling. In conclusion, modulating OGC expression in RPE affects EMT and mitochondrial function, making OGC a potential therapeutic target for subretinal fibrosis in nAMD.

## Graphical abstract

The diagram shows that OGC deficiency leads to mitochondrial dysfunction, which activates the PI3K/AKT/Slug pathway and triggers epithelial‐mesenchymal transition (EMT) in retinal pigment epithelial cells. This process promotes myofibroblast accumulation and subretinal fibrosis in laser‐induced CNV, contributing to the progression of AMD.

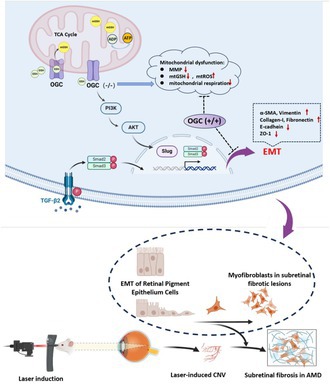

**Abbreviations**
AMD: age‐related macular degeneration
anti‐VEGF: anti‐vascular endothelial growth factor
CNV: choroidal neovascularization
EMT: epithelial‐mesenchymal transition
FFA: fluorescein angiography
GSH: glutathione
MMP: mitochondrial membrane potential
mtGSH: mitochondrial GSH
mtROS: mitochondrial reactive oxygen species
nAMD: neovascular AMD
OCR: oxygen consumption rate
OCT: optical coherence tomography
OGC: the oxoglutarate carrier
PS: phenylsuccinate
RCC: RPE‐choroid complex
RPE: retinal pigment epithelium
Slc25a11: solute carrier family 25 member 11
TFs: transcription factors
TGF‐β2: transforming growth factor‐beta2
α‐SMA: α‐smooth muscle actin

## 1 Introduction

Age‐related macular degeneration (AMD) is the leading cause of severe vision loss among the elderly, which is predicted to rise to 288 million affected individuals by the year 2040 (Wong et al. 2014). The wet form of AMD, or neovascular AMD (nAMD), is marked by the growth of new blood vessels in the macula (macular or choroidal neovascularization), and their exudation can cause severe vision impairment if untreated (Ambati and Fowler 2012). While anti‐vascular endothelial growth factor (anti‐VEGF) therapy has revolutionized nAMD treatment by effectively inhibiting CNV proliferation and reducing vascular leakage, nearly half of patients still experience poor visual outcomes, in part due to the development of subretinal fibrosis (Daniel et al. 2014). Therefore, the development of therapeutic strategies to specifically target and inhibit the progression of subretinal fibrosis necessitates a deeper understanding of the molecular mechanisms underlying its pathogenesis.

Subretinal fibrosis in nAMD is a complex fibrovascular structure with both blood vessels and fibrotic tissue, but the exact mechanism behind the transformation of abnormal vessels into fibrovascular tissue is unclear (Ishikawa, Kannan, et al. 2016; Matsuda et al. 2019; Zhang et al. 2025). Myofibroblasts, fibroblast‐like cells expressing α‐smooth muscle actin (α‐SMA), are the primary drivers of pathogenic fibrosis in nAMD (Shu and Lovicu 2017; Zhang et al. 2025). Increasing evidence suggests that the transdifferentiation of retinal pigment epithelial (RPE) cells into myofibroblasts via epithelial‐mesenchymal transition (EMT) is a critical process involved in subretinal fibrosis development (Ishikawa, Sreekumar, et al. 2016; Little et al. 2018; Liu, Zhang, et al. 2023; Shu et al. 2020). Transforming growth factor‐beta (TGF‐β) is a key regulator of fibrosis and induces EMT in RPE cells (Higashijima et al. 2022; Ishikawa, Sreekumar, et al. 2016; Shu et al. 2020). While the Smad signaling mediates TGF‐β‐induced EMT (Shu et al. 2020), other growth factors and cytokines, for example, IL‐2 (Jing et al. 2019), IL‐6 (Chen et al. 2020), epidermal growth factor, fibroblast growth factor, TNF‐α (Datlibagi et al. 2023), complement component C5a (Llorian‐Salvador et al. 2022), may also be involved. However, the precise molecular mechanisms and the critical regulator of this process remain to be fully elucidated.

The oxoglutarate carrier (OGC or Slc25a11) is an anion transporter that exchanges 2‐oxoglutarate for dicarboxylates across the inner mitochondrial membrane and transports glutathione (GSH) from the cytoplasm into the mitochondrial matrix, particularly in the heart, liver, brain, and kidney (Jang et al. 2021; Lash 2006; Putt et al. 2012; Ta et al. 2022; Wilkins et al. 2013; Zhong et al. 2008). Lash et al. first identified the role of OGC as a mitochondrial GSH (mtGSH) transporter (Chen and Lash 1998), and our lab further characterized it in human RPE cells (Sreekumar et al. 2021; Sreekumar et al. 2020; Wang et al. 2019). We confirmed OGC localization in hRPE cells and showed that its inhibition depletes mtGSH, impairs mitochondrial function, and increases apoptosis (Wang et al. 2019).

A growing body of evidence suggests mitochondrial dysfunction in the pathogenesis of AMD (Brown et al. 2019; Kaarniranta et al. 2020), but the specific role of OGC in regulating mitochondrial function and its contribution to RPE epithelial‐to‐mesenchymal transition (EMT) remain poorly understood. While the previous studies have highlighted the role of OGC in mitochondrial bioenergetics (Baulies et al. 2018; Buffet et al. 2018; Sreekumar et al. 2020), its contribution to subretinal fibrosis has not been investigated. We hypothesized that OGC deficiency impairs mitochondrial metabolism and redox homeostasis, creating a cellular environment that promotes RPE EMT, a key step in fibrotic progression. By investigating this link, we aim to identify novel metabolic targets to prevent or slow subretinal fibrosis. To address this, we used both in vitro and in vivo models to investigate the role of OGC and identified it as a key metabolic regulator of RPE EMT signaling in the retina.

## 2 Materials and Methods

### 2.1 Mice

The care, use, and treatment of experimental animals were in strict agreement with the ARVO Statement for the Use of Animals in Ophthalmic and Vision Research. All procedures involving animal care and experimentation were approved by the Institutional Animal Care and Use Committee (IACUC) of Eye & ENT Hospital, Fudan University, China. *OGC* (*Slc 25a11*) deficient (OGC^+/−^) mice (Cat. No. NM‐KO‐241583) were generated on a C57BL/6 background at Shanghai Model Organisms Center Inc. (Shanghai, China). Due to the embryonic lethality associated with the homozygous condition in mice (Lee et al. 2019), we used heterozygous (OGC^+/−^) mice in our studies. WT C57BL/6 mice were obtained from Shanghai Slac Laboratory Animal Co. Ltd. (Shanghai, China). All studies used mice aged 8–10 weeks, maintained on a standard laboratory chow in an air‐conditioned room with a 12‐h light/12‐h dark cycle. Additionally, all mice were screened for the rd8 mutation prior to the experiments.

### 2.2 Laser Induced Subretinal Fibrosis

We adapted the established mouse choroidal neovascularization (CNV) model with minor modifications for inducing subretinal fibrosis (Ishikawa, Sreekumar, et al. 2016). Mice were anesthetized using a cocktail of ketamine (90 mg/kg)/xylazine (10 mg/kg), and their pupils were dilated with atropine (1% w/v) and phenylephrine hydrochloride (2.5% w/v) (Covetrus, Portland, ME, USA). Four laser spots were generated by a 532‐nm laser at 120 mW for 0.1 s, with a spot size of 100 μm (Micron IV, Phoenix Research Laboratories, USA). Mice exhibiting post‐laser hemorrhaging were excluded from the study.

### 2.3 Optical Coherence Tomography (OCT), Fluorescein Angiography (FFA), and Choroidal Thickness

To comprehensively evaluate retinal structure and CNV leakage, color fundus imaging, optical coherence tomography (OCT), and fluorescein angiography (FFA) were performed on days 0, 7, 21, and 35 post‐laser. Briefly, mice were anesthetized, and their pupils were dilated, and images were acquired using a combined retinal imaging microscope and OCT device (Phoenix Research Laboratories, USA). Five minutes following intra‐peritoneal injection of 10% sodium fluorescein prepared in PBS (100 μL, #F6377, Sigma‐Aldrich), FFA was performed. Consistent exposure settings were used to ensure comparable data images between animals. Images were exported to ImageJ software (NIH, USA), and the borders of the CNV lesions in the FA images were outlined to quantify the area of fluorescein leakage by two independent investigators in a masked manner.

Choroidal thickness (ChT) measurements were performed as previously described (Tang et al. 2024). Briefly, mice were anesthetized, pupils dilated, and high‐resolution choroidal imaging was conducted using an ultra‐widefield swept‐source OCT (SS‐OCT) system (BM‐400K BMizar, TowardPi Medical Technology) at 400,000 A‐scans/s (*λ* = 1060 nm; axial resolution: 3.8 μm; transverse resolution: 10 μm). Radial scans (10° intervals) were acquired over a 12 × 12 × 6 mm volume centered on the optic nerve head (ONH). Only images with a quality score ≥ 8 (scale 1–10) were included. Imaging sessions were completed within 10 min to prevent corneal or lens opacification. Post‐imaging, eyes were rinsed with saline and treated with levofloxacin drops.

Choroidal thickness was measured from Bruch's membrane to the choroid–sclera interface. Twelve measurement points (T1–T6: temporal; N1–N6: nasal) were automatically registered by the OCT software at 0.15 mm intervals (0.15–0.90 mm from the ONH). Retinal thickness, choriocapillaris thickness, and large choroidal vessel layer thickness were manually segmented on pseudocolor OCT B‐scans. Total ChT was calculated as the sum of the capillary and large vessel layer thicknesses.

### 2.4 RPE‐Choroid Flat‐Mount Preparation and Measuring Area of CNV and Fibrosis

RPE‐choroid complex (RCC) flat mounts harvested at Days 7, 21, and 35 post‐laser (Ishikawa, Sreekumar, et al. 2016). In brief, the anterior segment, lens, and neural retina were removed, and the RPE‐choroid was fixed, followed by flattening. This was followed by incubation of samples overnight with FITC‐conjugated Isolectin‐B4 and collagen I (1:100 dilution). After incubation with the corresponding secondary antibody (1:100 dilution) for 30 min, followed by subsequent washing, the samples were mounted and visualized using a confocal microscope (Zeiss LSM510; Carl Zeiss, Germany). CNV and fibrotic lesion sizes were measured using area measurements of Isolectin‐B4 and collagen I staining on RCC flat mounts with ImageJ. Table 1 summarizes the antibodies used.

**TABLE 1:** Antibodies used in the study.

Antigen	Host species	Type	Dilution	Source
OGC (SLC25A11)	Rabbit	Monoclonal	1:1000 (WB)	Abcam
OGC (SLC25A11)	Mouse	Monoclonal	1:1000 (ELISA)	Santa Cruz Biotechnology
α‐SMA	Mouse	Monoclonal	1:1000 (WB) 1:100 (IF)	Abcam
E‐cadherin (24E10)	Rabbit	Monoclonal	1:1000 (WB) 1:100 (IF)	Cell Signaling
Snail	Rabbit	Monoclonal	1: 1000 (WB)	Cell Signaling
Slug	Rabbit	Monoclonal	1: 1000 (WB)	Cell Signaling
P‐SMAD2 (Ser465/467)/SMAD3 (Ser423/425)	Rabbit	Monoclonal	1: 1000 (WB)	Cell Signaling
SMAD2/3	Rabbit	Monoclonal	1: 1000 (WB)	Cell Signaling
PI3 Kinase p85	Rabbit	Monoclonal	1: 1000 (WB)	Cell Signaling
pPI3 Kinase p85 Kinase	Rabbit	Monoclonal	1: 1000 (WB)	Cell Signaling
pAKT (ser473)	Rabbit	Monoclonal	1: 1000 (WB)	Cell Signaling
AKT	Rabbit	Monoclonal	1: 1000 (WB)	Cell Signaling
p‐mTOR (Ser2448)	Rabbit	Monoclonal	1: 1000 (WB)	Cell Signaling
mTOR	Rabbit	Monoclonal	1: 1000 (WB)	Cell Signaling
p‐(ERK1/2) (Thr202/Tyr204)	Rabbit	Monoclonal	1: 1000 (WB)	Cell Signaling
ERK1/2	Rabbit	Monoclonal	1: 1000 (WB)	Cell Signaling
p‐GSK‐3β (Ser9)	Rabbit	Monoclonal	1: 1000 (WB)	Cell Signaling
GSK‐3β	Rabbit	Monoclonal	1: 1000 (WB)	Cell Signaling
TGF‐β2	Rabbit	Polyclonal	1: 1000 (WB)	Proteintech
α‐Tubulin	Rabbit	Polyclonal	1: 1000 (WB)	Cell Signaling
GAPDH	Mouse	Monoclonal	1:1000 (WB)	Abcam

### 2.5 Histology Analysis

Eyes were enucleated, and the anterior segment and lens were removed. The posterior eye cups were fixed overnight in 4% PFA, then paraffin‐embedded, and 5 μm sections were cut and stained with hematoxylin and eosin. The stained sections were imaged using an Olympus SL120 Virtual Microscopy Slide Scanner (20× objective, Olympus, USA).

### 2.6 Cell Culture and Treatment

ARPE‐19 cells (Shanghai Cell Bank of the Chinese Academy of Sciences, Shanghai, China) were cultured in DMEM/F‐12 supplemented with 10% fetal bovine serum and 1% penicillin–streptomycin (Thermo Fisher Scientific, USA). ARPE‐19 cells were utilized only up to passage 5 following receipt to ensure cellular integrity and phenotype consistency (Pfeffer and Fliesler 2022). To investigate the effect of OGC inhibition on EMT markers, cells were incubated with the OGC inhibitor phenylsuccinic acid (PS) (5 mM; Sigma‐Aldrich, USA) at varying doses (2, 5, 10 mM). For performing studies following OGC modulation, cells with either OGC knockdown or overexpression were treated with or without TGF‐β2 (10 ng/mL, Sigma‐Aldrich) for 48 h.

### 2.7 Transfection and Real‐Time Quantitative RT‐PCR

ARPE‐19 cells were cultured to 70% confluence and transfected with OGC or control siRNAs (25 nM, Sangon Biotech, China) for 24 h, as described previously (Wang et al. 2019). The specific siRNA sequences are listed in Table 2. For OGC overexpression, cells were transfected with the pCMV6‐AC‐GFP plasmid containing human OGC or an empty vector using Lipofectamine 3000. After 24 h, total RNA was extracted for RT‐qPCR to assess mRNA expression changes, with relative expression calculated using 2^−ΔΔCT^. Primer sequences used for RT‐PCR are listed in Table 2.

**TABLE 2:** OGC siRNA knockdown sequences and OGC overexpression constructs.

Gene	Forward (5′–3′)	Reverse (5′–3′)
siOGC#1	AGCCGGAATACAAGAACGGG	CGTCCTAGACACAGACAGGC
siOGC#2	TGATCAGCGGTCTTGTCACC	CATGAGAGACCGAAAGGGCA
siSLUG#1	CCCAUUCUGAUGUAAAGAATT	UUCUUUACAUCAGAAUGGGTT
siSLUG#2	GAAUGUCUCUCCUGCACAATT	UUGUGCAGGAGAGACAUUCTT
siSMAD2#1	GAAUUGAGCCACAGAGUAA	UUACUCUGUGGCUCAAUUC
siSMAD2#2	GGAUUGAACUUCAUCUGAA	UUCAGAUGAAGUUCAAUCC
siSMAD3#1	GCUUGGUGAAGAAGCUCAA	UUGAGCUUCUUCACCAAGC
si SMAD3#2	CCAGAGCAAUAUUCCAGAA	UUCUGGAAUAUUGCUCUGG
si NC	UUCUCCGAACGUGUCACGU	ACGUGACACGUUCGGAGAA
pCMV‐AC‐OGC #1	CCCAGTCACGACGTTGTAAAACG	CAGGAAACAGCTATGAC
pCMV‐AC‐OGC #2	CAUUAGUGAUGAAGAGGAATT	UUCCUCUUCAUCACUAAUGGG
pCMV‐AC‐control	GCTGTAGGAACCGCCGCCGTGTC	UUGUGCAGGAGAGACAUUCTT

### 2.8 Western Blot Analysis

After treatment, ARPE‐19 cells were washed with PBS, and protein samples were extracted using RIPA lysis and extraction buffer containing a protease inhibitor cocktail (Thermo Scientific, MA, USA). Cells were kept on ice for 5 min, followed by centrifugation at ~14,000×*g* for 15 min. The protein concentration in the supernatant was then quantified using the Bio‐Rad protein assay (Bio‐Rad, Hercules, CA, USA) (Wang et al. 2019). Equal protein amounts (20 μg) were subjected to western blot analysis, and proteins were transferred to a PVDF membrane (Millipore, USA). Membranes were blocked, probed with primary antibodies (Table 1), incubated with secondary antibodies, washed, and visualized using chemiluminescent substrate (Millipore). Alpha‐tubulin/GAPDH served as a loading control, and protein bands were quantified with ImageJ software (Wang et al. 2019).

### 2.9 Enzyme‐Linked Immunosorbent Assay (ELISA)

ELISA was performed on protein extracts from the RPE/choroid complex isolated from Day 35 post‐lasered WT (8–10 weeks‐old) mice. The RPE/choroid complexes were homogenized in 300 μL extraction buffer (ab19397, Abcam) and protein concentration was measured. Microtiter plate (Sigma Aldrich, M9410‐1CS) was coated with target protein (OGC, 1:1000 dilution) overnight, washed and blocked with BSA (5%) for 2 h at RT. Equal amounts of protein (50 μg) were used for ELISA. Following incubation with secondary antibody (1:10,000 dilution; KPL, 074–1506) and reagent addition, absorbance was measured at 450 nm using a microplate reader.

### 2.10 Isolation of Mitochondrial and Cytosolic Fractions and Assay for GSH

Mitochondrial and cytosolic fractions of ARPE‐19 cells were isolated using a kit (ab65320, Abcam), and protein concentration was measured. Equal amounts of protein were loaded into a 96‐well plate, and GSH concentration was measured using a colorimetric assay (ab65322, Abcam) (Sreekumar et al. 2020). Total GSH levels were normalized to protein content and expressed as a percent relative to the average of control samples.

### 2.11 Cell Migration and Wound Healing

For wound‐healing assay, ARPE‐19 cells (2 × 10^5^/well) were seeded in a 24‐well plate and starved overnight in FBS‐free medium. After different treatments, a pipette tip (200 μL) was used to create a scratch on the bottom of the dish (Ma et al. 2023). Wound areas were recorded at 0‐, 24‐, and 48‐h post‐scratch using an inverted microscope (Olympus) and analyzed with ImageJ software.

Transwell migration assays were performed using a modified Boyden chamber model (Transwell apparatus, 8.0 μm pore size, Costar) as per the manufacturer's protocol. For RPE cell migration, the lower chamber was coated with fibronectin (0.3 mg/mL) for 30 min and filled with 0.6 mL of serum‐free medium. OGC knockdown or overexpression RPE cells (5 × 10^4^ cells, 200 μL) were plated in the upper chamber and treated with or without TGF‐β2 (10 ng/mL). After 5 h, non‐migrant cells were removed, and migrated cells were fixed with 4% PFA, stained with 0.5% toluidine blue, and counted in three random fields per chamber using a bright‐field microscope (Olympus). The wound area averages were calculated using ImageJ.

### 2.12 Effect of OGC Silencing or Overexpression on TGFβ Induced Cell Proliferation

ARPE cells (5 × 10^3^ cells/well) were transfected with either OGC siRNA, OGC overexpression plasmid, or appropriate controls. After 24 h, cells were treated with TGF‐β2 (10 ng/mL) for an additional 48 h in FBS‐free medium. Prior to the end of the treatment, CCK‐8 solution (10 μL, Beyotime Biotechnology) was added to each well and incubated for 2 h at 37°C. Cell proliferation was quantified by measuring absorbance at 450 nm using a microplate reader.

### 2.13 Detection of Mitochondrial Membrane Potential (MMP) and mtROS

ARPE‐19 cells were transfected with either OGC siRNA or an OGC overexpression plasmid. After 24 h of transfection, the cells were treated with TGF‐β2 (10 ng/mL) for 48 h. Mitochondrial membrane potential (MMP) was assessed using a tetramethylrhodamine ethyl ester (TMRE) Mitochondrial Membrane Potential Assay Kit (C2001S, Beyotime Biotechnology, China), as previously described (Lan et al. 2024). Briefly, cells were incubated with TMRE at a 1X final concentration at 37°C for 35 min. Fluorescent images were captured using a fluorescence microscope, and fluorescence intensity was quantified using ImageJ software.

Mitochondrial superoxide production was measured using MitoSOX Red following the manufacturer's protocol (#M36008, Invitrogen, USA). Briefly, cells were incubated with 5 μM MitoSOX solution at 37°C for 20 min in the dark. After incubation, cells were washed three times with PBS, and fluorescent signals were captured using a Zeiss LSM510 confocal microscope. Images were quantified using ImageJ, and fluorescence intensity was normalized to control values and expressed as a percentage of the control.

### 2.14 Measurement of Mitochondrial Respiration

Mitochondrial bioenergetics were determined by measuring the oxygen consumption rate (OCR) of ARPE‐19 cells using a Seahorse XFe96 Analyzer (Agilent, Santa Clara, CA, USA) following established protocols (Sreekumar et al. 2020). Cells at approximately 70% confluence were transfected with either OGC siRNA or OGC overexpression vector for 48 h, then seeded at 10,000 cells per well in Seahorse XF96 tissue culture plates. After 24 h, cells were treated with TGF‐β2 (10 ng/mL) for 48 h and assayed for mitochondrial function. Basal respiration, ATP generation, maximal respiration, and spare respiratory capacity were measured. After the assay, cells were lysed, protein concentration was determined, and data were normalized to protein content (μg protein).

### 2.15 Statistical Analysis

All experiments were performed at least three times. Quantitative data were presented as mean ± standard deviation, and the one‐way ANOVA followed by Tukey post‐test was conducted to analyze data differences (GraphPad Prism, version 5; GraphPad Software Inc., La Jolla, CA, USA). *p* < 0.05 was considered statistically significant.

## 3 Results

### 3.1 Aggravation of Subretinal Fibrosis in OGC ^+/−^ Mice

**FIGURE 1:**
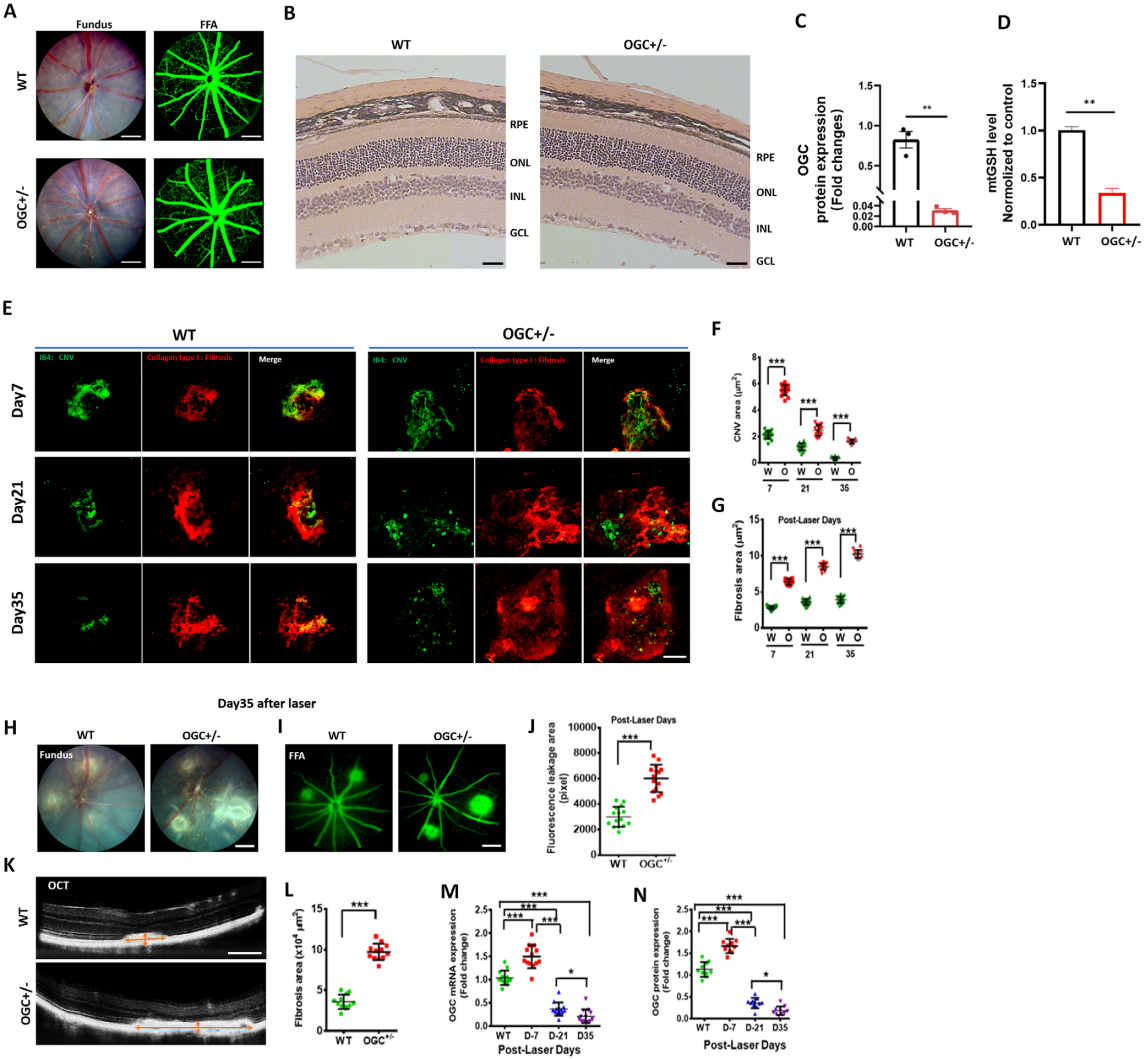
Subretinal fibrosis was significantly augmented in OGC^+/−^ mice. (A) Fundus and fluorescein angiography (FFA) of WT and OGC+/− mice. Scale bar: 300 μm. (B) Retinal histology (hematoxylin and eosin staining) of the retina in WT and OGC^+/−^ mice. Scale bar: 20 μm. No major changes in fundus, FFA or histology were observed in the OGC^+/−^ mice. (C) Protein expression levels of OGC were significantly decreased in the RPE‐choroid complexes of the OGC^+/−^ mice. (D) Mitochondrial GSH (mtGSH) levels were reduced by 67% in the RPE‐choroid complexes of OGC^+/−^ mice compared to WT mice. (Mean ± SD (*n* = 3)), Student's *t*‐test. **p < 0.01. (E) RPE/choroid flat mounts were stained for isolectin B4 (green) and collagen type I (red) to represent CNV and subretinal fibrosis, respectively, on Days 7, 21, and 35 post‐laser in WT and OGC^+/−^ mice. Scale bar: 100 μm. (F, G) Quantitative measurement of CNV and fibrosis lesion area on Days 7, 21, and 35 after laser. Mean ± SD, *n* = 6 eyes per group, 15–20 lesions per group (***p < 0.001). Fundus (H) and fluorescein angiography (FFA) (I) as well as leakage quantification (J) were performed on Day 35 post‐laser (scale bar: 300 μm). FFA quantification (J) showed a significantly increased area of diffuse hyperfluorescence and leakage in OGC+/− mice compared to WT (***p < 0.001). Optical coherence tomography (OCT) (K) and fibrosis quantification from OCT (L) on Day 35 post‐laser (scale bar: 200 μm). Quantification of OCT images revealed a significantly increased subretinal fibrosis area in OGC^+/−^ mice (***p < 0.001). mRNA (M) and protein (N) expression levels of OGC in the RPE‐choroid complexes of WT mice following laser injury were significantly decreased on Days 21 and 35 post‐laser (*p < 0.05; **P < 0.01; ***p < 0.001).

A genetically engineered mouse model targeting OGC (*Slc25a11*) was used to study the role of the transporter in subretinal fibrosis. Comprehensive analysis, including fundus examination, fluorescein angiography (FFA), histology, OCT, as well as measurements of choroidal capillary length and choroidal thickness in 8–10‐week‐old OGC^+/−^ mice, revealed no notable retinal phenotype (Figure 1A,B, Figure S1A–F). RT‐PCR and western blot analysis indicated that mRNA and protein expression levels of OGC were significantly decreased in the RPE‐choroid complexes of OGC^+/−^ mice, with a ~50% decrease versus WT mice (Figure 1C, Figure S1G). Mitochondrial GSH levels in the RPE‐choroidal tissue were significantly lower (67%) in OGC^+/−^ mice compared to WT (*p* < 0.01) (Figure 1D), whereas GSH levels in whole cell lysates from the RPE‐choroid complex did not differ significantly between OGC^+/−^ and WT mice (Figure S1H). These results are consistent with our previous study (Wang et al. 2019) that further supports its role in mitochondrial GSH uptake.

Next, we sought to determine how OGC deficiency influences the progression of CNV and subretinal fibrosis following laser injury. To assess the temporal progression of CNV and fibrosis following laser irradiation, RPE/choroid flat mounts were immunostained with isolectin B4 (CNV marker, green) and collagen type I (fibrosis marker, red). CNV lesion size peaked at Day 7 post‐laser and gradually regressed, nearly disappearing by Day 35 (Figure 1E, Figure S1I). CNV area was almost three times larger in OGC^+/−^ mice compared to WT at Day 7 (*p* < 0.001) (Figure 1F). Fibrosis progression showed a gradual increase from Day 7, with maximal subretinal fibrosis size from Days 21 to 35. In OGC^+/−^ mice, subretinal fibrosis lesions were significantly larger at Days 7, 21, and 35 post‐laser, with a contrasting trend to WT mice, where fibrosis regressed from Day 21 (Figure 1G). Fundus and FFA analysis revealed significantly increased lesion size and diffuse hyperfluorescence and leakage in OGC^+/−^ mice compared to WT (Figure 1H,I,J). OCT examination also showed a significantly larger lesion area in OGC^+/−^ mice at day 35 post‐laser (Figure 1K,L).

To further explore whether OGC expression is dynamically regulated in response to laser injury, we assessed the mRNA and protein expression levels of OGC over time following laser irradiation in the RPE‐choroid complex of WT mice. Our results revealed a significant upregulation of OGC mRNA and protein expression on Day 7 post‐laser, followed by a notable decrease at Days 21 and 35 (Figure 1M,N). These findings suggest that OGC levels decline during subretinal fibrosis formation, and the deficiency of OGC may significantly exacerbate the subretinal fibrosis observed.

### 3.2 TGF‐β2‐Induced EMT in RPE Cells Was Exacerbated by OGC Inhibition but Attenuated by OGC Overexpression

**FIGURE 2:**
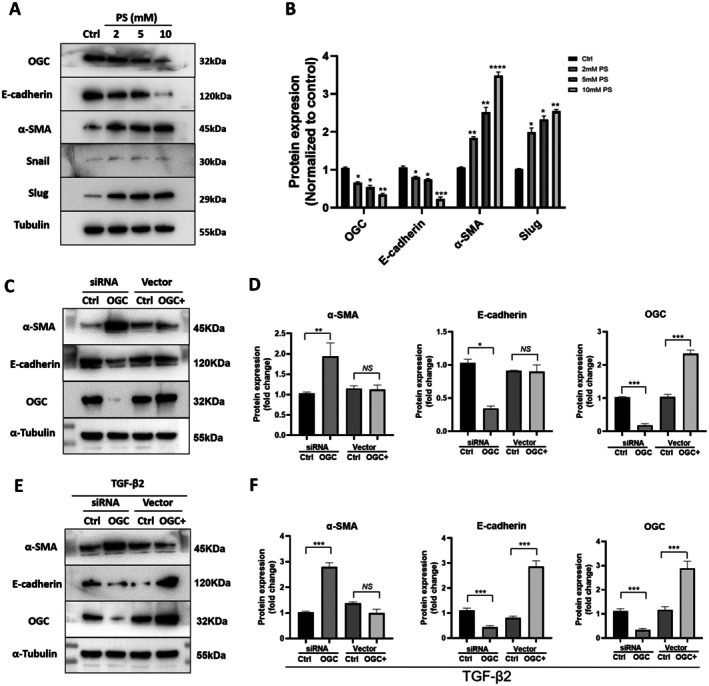
OGC inhibition aggravated EMT, whereas overexpression attenuated TGF‐β2‐induced EMT. ARPE‐19 cells were treated with varying doses of PS (0, 2, 5, 10 mM) for 48 h. Samples were analyzed for EMT markers via western blot. (A) Representative western blots of E‐cadherin, α‐SMA, Snail, Slug, and OGC at varying PS doses. (B) Gel quantification from panel A, showing fold changes normalized to α‐Tubulin. (C–F) OGC silenced or over expressed (OGC+), ARPE‐19 cells were stimulated with TGF‐β2 (10 ng/mL) for 48 h. Protein expression of E‐cadherin, α‐SMA, OGC, and α‐Tubulin in ARPE‐19 cells treated with or without TGF‐β2, and the quantification. Data represent mean ± SD (*n* = 3). *p < 0.05; **p < 0.01; ***p < 0.001; ****p < 0.0001; NS, not significant.

We and others have demonstrated that in late‐stage neovascular AMD, RPE cells lose their characteristic epithelial features and acquire mesenchymal features, a hallmark of epithelial–mesenchymal transition (EMT) (Hirasawa et al. 2011; Ishikawa, Kannan, et al. 2016; Ishikawa, Sreekumar, et al. 2016; Sreekumar et al. 2025). To explore whether OGC plays a functional role in RPE EMT, we first examined the effect of pharmacological inhibition of OGC on EMT marker expression. ARPE‐19 cells were treated with varying concentrations phenylsuccinate (PS) (0, 2, 5, and 10 mM), a pharmacological inhibitor OGC (Wang et al. 2019) for 48 h. PS treatment induced a dose‐dependent upregulation of mRNA expression of mesenchymal markers (α‐SMA, Collagen‐I, fibronectin) and a downregulation of the epithelial marker E‐cadherin, with the highest dose (10 mM) causing a 3.4‐fold increase in α‐SMA protein expression (*p* < 0.0001) and an ~80% decrease in E‐cadherin protein expression (*p* < 0.001) (Figure S2A). PS also significantly increased the protein expression of the EMT markers and transcription factor Slug (Figure 2A,B). As expected, PS‐ treatment also downregulated OGC expression in a dose‐dependent manner (Figure 2A,B), consistent with previous findings (Wang et al. 2019).

To corroborate the findings obtained with the OGC pharmacological inhibitor PS, we used genetic manipulation strategies involving OGC knockdown and overexpression in RPE cells. Knockdown of OGC by siRNA significantly upregulated α‐SMA (*p* < 0.01) and decreased E‐cadherin (*p* < 0.05) expression at mRNA (Figure S2B) and protein levels (Figure 2C,D). Notably, OGC overexpression alone did not significantly affect the expression of these EMT markers (Figure 2C,D). Given that TGF‐β2 is the predominant TGF‐β isoform in the posterior segment of the eye and a potent inducer of RPE EMT (Hachana and Larrivee 2022; Shu et al. 2020), we investigated its interaction with OGC. Cotreatment with TGF‐β2 (10 ng/mL for 48 h) in OGC silenced cells further decreased E‐cadherin expression and increased α‐SMA expression (Figure 2E,F). However, OGC overexpression completely abrogated the TGF‐β2‐induced EMT response as evidenced by the restoration of E‐cadherin and suppression of α‐SMA (Figure 2E,F). Notably, TGF‐β2 treatment alone significantly inhibited OGC protein expression (Figure S3A,B). In contrast, overexpression of OGC appeared to reverse EMT in response to TGF‐β2 stimulation. Immunofluorescence staining further supported these findings, revealing that OGC deficiency exacerbated TGF‐β2‐induced EMT, while OGC overexpression reversed this effect (Figure S3C). We also observed pronounced EMT‐associated morphological changes in OGC‐silenced ARPE‐19 cells compared to control cells (Figure S3D, *black arrows*). These morphological alterations were most prominent in the co‐treatment group (TGF‐β2 + OGC silencing), demonstrating a clear transition toward a spindle‐shaped, fibroblast‐like, mesenchymal phenotype. Collectively, these data strongly support a critical role for OGC in regulating EMT in RPE cells.

### 3.3 OGC Regulates TGF‐ β2‐Induced Proliferation and Migration of RPE

**FIGURE 3:**
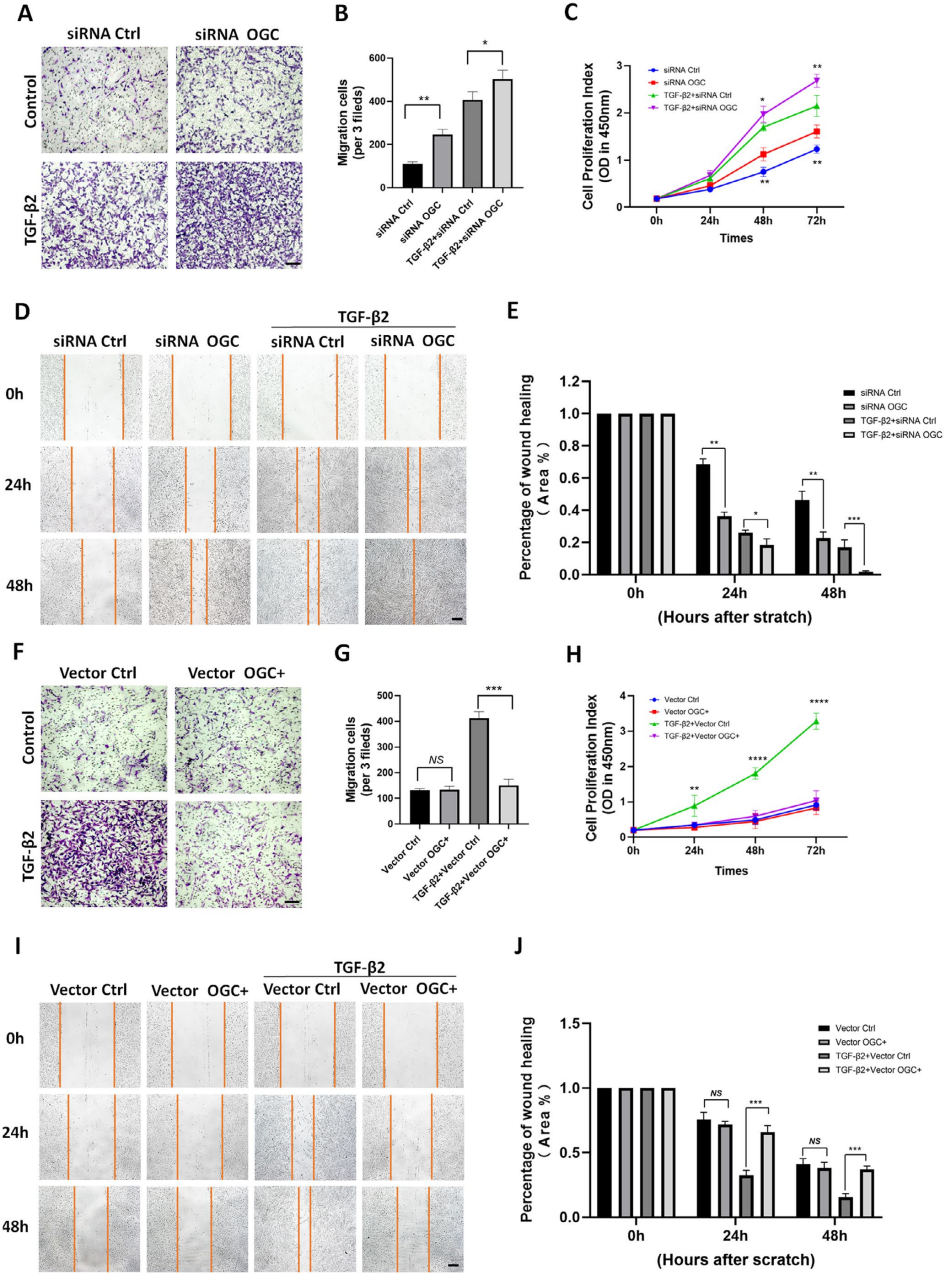
Silencing of OGC aggravates TGF‐β2‐induced cell proliferation and migration, which was inhibited by OGC overexpression. ARPE‐19 cells were transfected with control siRNA (Ctrl) and OGC siRNA, empty (Ctrl) vector, and OGC‐encoding (overexpressed) vector (OGC+), with or without TGF‐β2 treatment for 48 h. (A) Transwell migration assays were performed in OGC‐silenced cells treated with or without TGF‐β2 (10 ng/mL) for 48 h (scale bar: 200 μm). (B) The bar graph depicts the average number of cells migrated per field. (C) Cell proliferation in OGC‐silenced cells with or without TGF‐β2 (10 ng/mL) treatment at 24, 48, and 72 h (*p < 0.05, **p < 0.01). (D) Scratch assays were performed in OGC‐silenced cells with or without TGF‐β2 (10 ng/mL) treatment for 48 h (scale bar: 200 μm). (E) The quantification of migrated cells to the scratched area at 24 and 48 h after wounding (*p < 0.05, **p < 0.01, ***p < 0.001). (F) Transwell migration assays were performed in OGC overexpressed cells with or without TGF‐β2 (10 ng/mL) treatment for 48 h (scale bar: 100 μm). (G) The bar graph shows the average number of cells migrated per field. (H) Cell proliferation in OGC overexpressed cells with or without TGF‐β2 (10 ng/mL) treatment at 24, 48, and 72 h. (I) Scratch assays were performed in OGC overexpressed ARPE‐19 cells with or without TGF‐β2 (10 ng/mL) treatment up to 48 h (scale bar: 200 μm). (J) The quantification of migrated cells to the scratched area at 24 and 48 h after wounding. Data are presented as mean + SD, *n* = 3 per group, NS, not significant; *p < 0.05, **p < 0.01, ***p < 0.001.

To assess the functional relevance of OGC in TGF‐β2‐induced EMT, we examined whether OGC silencing or overexpression modulates two key EMT‐associated behaviors: RPE cell proliferation and migration. OGC silencing enhanced cell proliferation (*p* < 0.01) and migration (32% and 45% at 24 and 48 h, *p* < 0.001, *p* < 0.001) and wound closure (*p* < 0.01), with TGF‐β2 co‐treatment further amplifying these effects (Figure 3A–E). OGC overexpression significantly reduced TGF‐β2‐induced RPE migration, decreasing cell migration by approximately 53% at 48 h (*p* < 0.001; Figure 3F,G), with similar results in Transwell migration assays and delayed wound closure (Figure 3F–J). Additionally, OGC silencing induced RPE cell proliferation, which was further significantly increased with TGF‐β2 treatment at 48 and 72 h (*p* < 0.01, *p* < 0.01) (Figure 3C). In contrast, OGC overexpression significantly reduced TGF‐β2‐induced proliferation (*p* < 0.0001) (Figure 3H). These results suggest a strong association between OGC expression and TGF‐β2‐induced migration and proliferation.

### 3.4 OGC Deficiency Exacerbates TGF‐β2‐Induced Mitochondrial Dysfunction in RPE Cells

**FIGURE 4:**
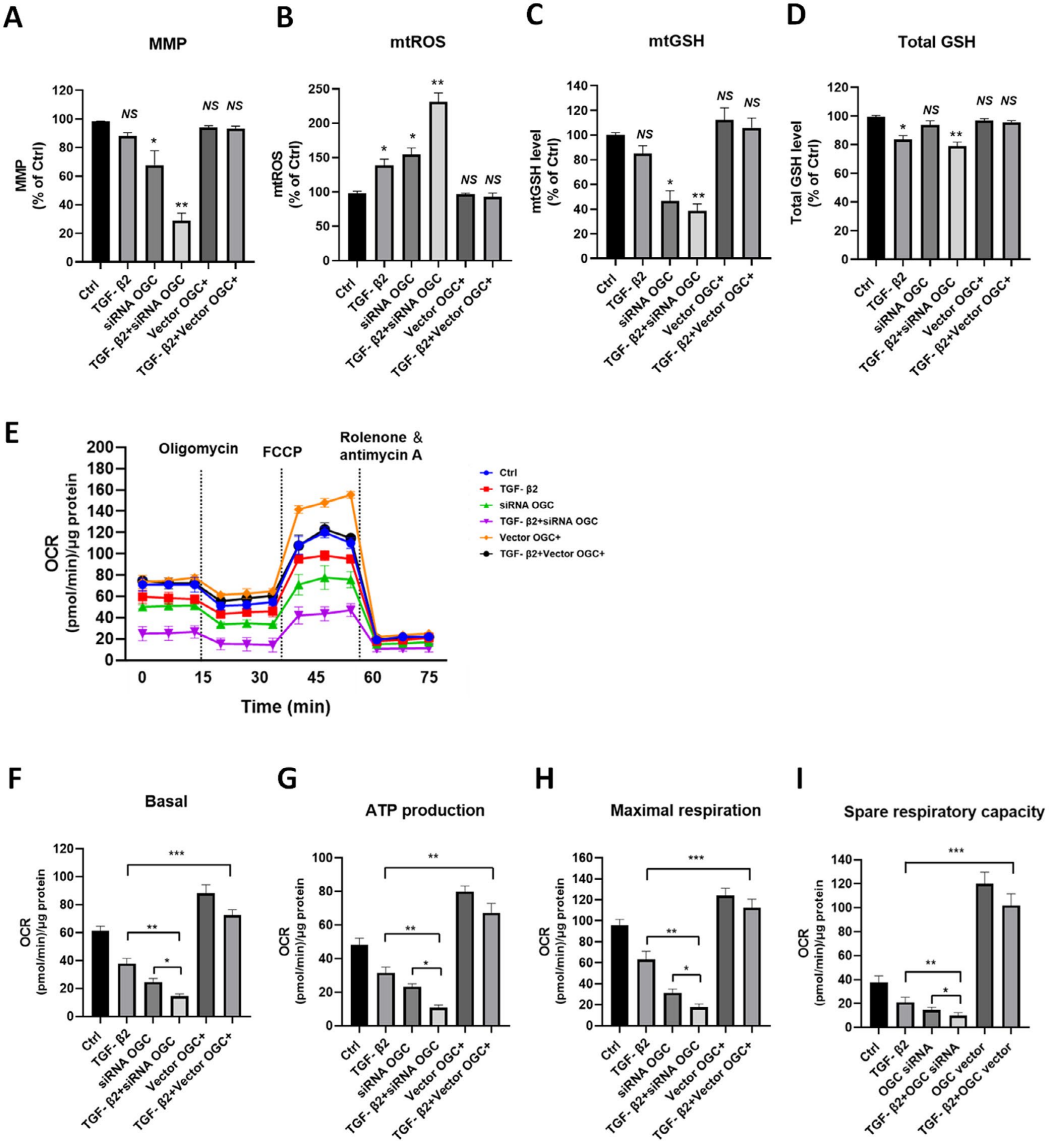
OGC depletion impairs mitochondrial function and mitochondrial bioenergetics while overexpression reversed these effects. ARPE‐19 cells were transfected to either knockdown or overexpress OGC and treated with TGF‐β2 (10 ng/mL) for 48 h. The following were studied: Mitochondrial membrane potential (MMP) (A), the mitochondrial ROS (mtROS) (B), mitochondrial GSH (mtGSH) (C), and total GSH levels (D). Mitochondrial bioenergetics were analyzed using Seahorse XFe96 (E–I). OGC silencing reduces mitochondrial bioenergetic parameters (basal respiration, ATP production, maximal respiration, and spare respiratory capacity), while OGC overexpression restored these parameters. OGC inhibition significantly reduced mitochondrial respiration, an effect further aggravated by TGF‐β2 treatment. Data were normalized to μg/cellular protein. Values are means ± SD. *n* = 9–15. NS, nonsignificant difference, *p < 0.05, **p < 0.01, ***p < 0.001.

Given OGC's established role in mitochondrial GSH transport (Sreekumar et al. 2020; Wang et al. 2019), we investigated whether OGC deficiency impairs mitochondrial antioxidant capacity in RPE cells undergoing EMT. Silencing OGC resulted in a ~22% reduction in mitochondrial membrane potential (MMP) and a significant increase in mitochondrial reactive oxygen species (mtROS) production (*p* < 0.05, *p* < 0.05). Co‐treatment with TGF‐β2 further worsened these effects, causing a 40% decrease in MMP and a marked increase in mtROS (*p* < 0.01, *p* < 0.01) (Figure 4A,B). Analysis of mtROS showed a 0.5‐fold increase in OGC‐silenced cells compared to controls (*p* < 0.05). TGF‐β2 co‐treatment further exacerbated this effect, leading to a 2.3‐fold increase in mtROS levels (*p* < 0.01) (Figure 4B).

To determine whether these mitochondrial changes were linked to GSH imbalance, we next assessed mtGSH levels. OGC silencing led to a significant reduction in mtGSH, with a 62% decrease in OGC‐silenced cells and a 78% decrease in TGF‐β2 co‐treated OGC‐silenced cells (*p* < 0.05, *p* < 0.01) (Figure 4C), while total cellular and cytosolic GSH levels remained unaffected (Figure 4D). Given the observed changes in mtGSH upon OGC knockdown, we next investigated their potential impact on mitochondrial bioenergetics. OGC knockdown resulted in decreased basal respiration, ATP production, maximal respiration, and spare respiratory capacity, with the most pronounced effects observed in TGF‐β2 co‐treated cells (Figure 4E–I). Finally, to determine whether restoring OGC expression could rescue mitochondrial health, we evaluated mitochondrial bioenergetic parameters in OGC‐overexpressing cells exposed to TGF‐β2. OGC overexpression reduced TGF‐β2‐induced mtROS generation and restored mitochondrial function by increasing mtGSH levels (Figure 4E–I). These results suggest that OGC deficiency leads to decreased mtGSH, elevated mtROS, and mitochondrial dysfunction, potentially contributing to the EMT phenotype in RPE cells.

### 3.5 OGC Deficiency Promotes RPE Cell Proliferation and Migration via Slug‐Dependent EMT

**FIGURE 5:**
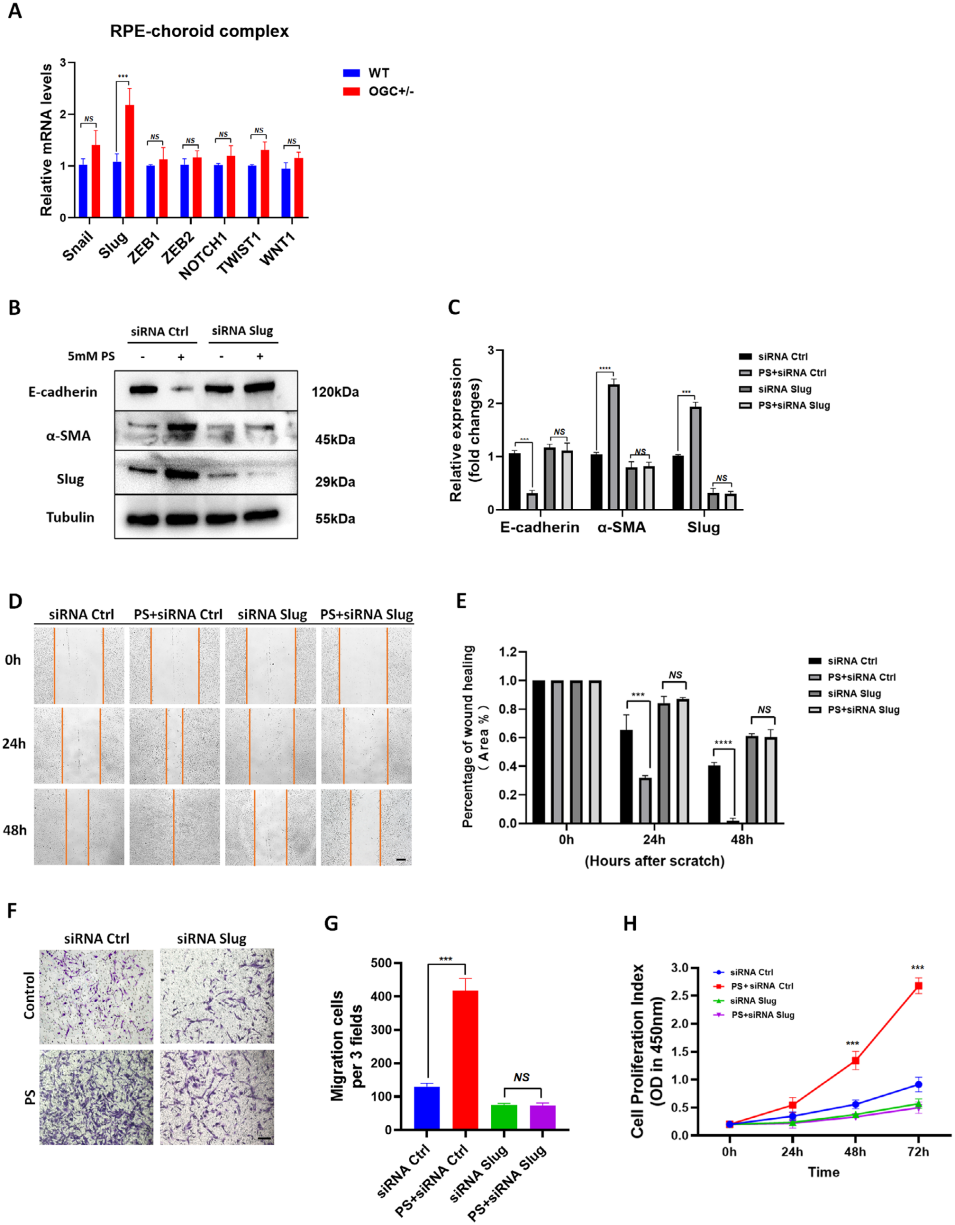
Loss of OGC facilitates RPE cell proliferation and migration through Slug‐mediated EMT. (A) The mRNA expression of seven epithelial‐mesenchymal transition‐regulating transcription factors (EMT‐TFs) in the RPE/choroid complex of WT and OGC^+/−^ mice. Data normalized to GAPDH is shown. (B, C) ARPE‐19 cells were transfected with Slug siRNA and treated with or without OGC inhibitor (PS). Protein expression of Slug was significantly upregulated in OGC‐deficient cells. Knockdown of Slug significantly inhibited EMT induction with OGC deficiency. (H) Cell proliferation and (D–G) migration were significantly reduced with Slug knockdown. All data are shown as mean ± SD. *n* = 3 per group. NS, not significant; *p < 0.05, **p < 0.01, ***p < 0.001. (D) scale bar: 200 μm, (F) scale bar: 100 μm.

To determine the mechanism by which OGC deficiency promotes EMT, we examined whether key EMT‐associated transcription factors are differentially regulated. Snail and Slug are the transcription factors (TFs) that repress E‐cadherin while stimulating α‐SMA, known to induce EMT in RPE cells in vitro (Ishikawa, Sreekumar, et al. 2016; Little et al. 2018). We evaluated the mRNA expression of key EMT‐TFs in the RPE‐Choroid complex of OGC^+/−^ mice compared to WT mice. RT‐qPCR showed a significant increase in Slug, but not Snail, mRNA in OGC^+/−^ mice (*p* < 0.001) (Figure 5A). Slug expression also increased in ARPE‐19 cells with higher PS concentrations (Figure 2A,B). These results suggest that OGC deficiency promotes EMT in RPE cells, potentially through Slug upregulation. To test this hypothesis, Slug was knocked down in ARPE‐19 cells treated with or without PS, and EMT markers were examined. Western blot analysis showed that PS‐induced EMT (downregulation of E‐cadherin and upregulation of α‐SMA) was inhibited by Slug knockdown (Figure 5B,C), indicating Slug is required for OGC inhibition‐induced EMT. We next examined whether Slug also mediates the enhanced migration and proliferation observed with OGC inhibition. Scratch and Transwell assays demonstrated that PS (5 mM) for 48 h significantly promoted ARPE‐19 cell migration, which was blocked by Slug knockdown (Figure 5D–G). Slug silencing also reduced PS‐induced increases in cell proliferation at 48 and 72 h (*p* < 0.001; Figure 5H). Together, these results demonstrate that OGC deficiency promotes RPE cell EMT, proliferation, and migration in a Slug‐dependent manner, highlighting Slug as a key downstream effector of OGC‐regulated EMT.

### 3.6 OGC Deficiency Induces EMT Through pSmad2/3 and PI3K/AKT Signaling

**FIGURE 6:**
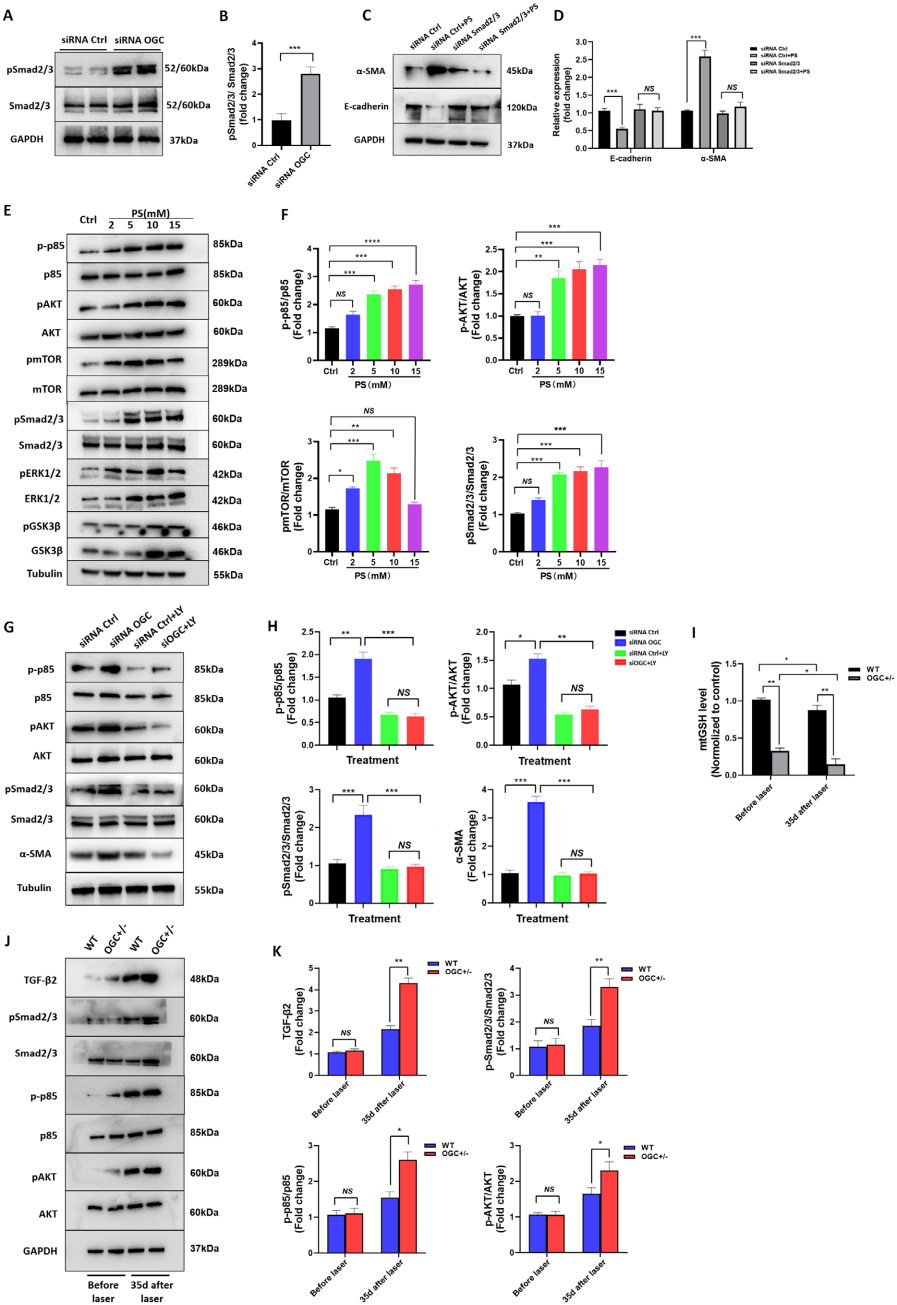
OGC depletion promotes EMT via upregulation of pSmad2/3‐dependent PI3K/AKT signaling pathway activation. (A, B) Western blot analysis and quantification of phosphorylated Smad2/3 in OGC silenced ARPE‐19 cells. (C, D) Western blot analysis and quantification α‐SMA and E‐cadherin expression in Smad2/3 silenced ARPE‐19 cells, with or without treatment with the OGC inhibitor, PS. (E, F) ARPE‐19 cells were treated with various doses of the OGC inhibitor (PS), and cell lysates were analyzed by western blot analysis of Phospho‐PI3 Kinase p85 (p‐P85), PI3 Kinase p85 (p85), p‐AKT, AKT, p‐mTOR, mTOR, pERK1/2, ERK1/2, pGSK3β, and GSK3β. (G, H) ARPE‐19 cells were transfected with control siRNA or OGC siRNA for 48 h, followed by treatment with vehicle alone or LY294002 (10 μM, an inhibitor of phosphatidylinositol 3‐kinase) for 1 h. LY294002 significantly inhibited the activation of PI3 Kinase p85 and AKT in OGC deficient cells. Data shown are mean ± SD, *N* = 3, NS, not significant; *p < 0.05, **p < 0.01, ***p < 0.001, ****p < 0.0001. (I) Mitochondrial GSH (mtGSH) levels were measured in the RPE‐choroid complexes of both non‐lasered and 35‐day post‐lasered WT and OGC^+/−^ mice. A significant reduction in mtGSH was observed in OGC^+/−^ mice compared to WT under non‐lasered conditions (**p < 0.01), and levels were further decreased 35 days after laser treatment (*p* < 0.05). Data represents SD (*n* = 3; Student's *t*‐test; *p < 0.05, **p < 0.01). (J, K) Western blot analysis of the RPE/choroid complex from WT and OGC^+/−^ mice before and 35 days after laser treatment. Protein expression levels of TGF‐β2, phosphorylated SMAD, AKT, and p85 were significantly increased in the RPE/choroid complexes of OGC^+/−^ mice compared to WT mice at 35 days post‐laser. Data are presented as mean ± SD (*n* = 3); Student's *t*‐test (*p < 0.05, **p < 0.01; NS = not significant).

EMT is regulated by multiple signaling pathways that converge on transcription factors such as Snail and Slug. To identify the downstream mechanisms involved in OGC deficiency–induced EMT, we first examined activation of the canonical TGF‐β/Smad signaling cascade. Western blot analysis revealed a significant increase in phosphorylated Smad 2/3 levels (*p* < 0.001, Figure 6A,B) in OGC deficient cells. Knockdown of Smad2/3 resulted in a notable decrease in α‐SMA expression in PS treated‐RPE cells (Figure 6C,D), suggesting that Smad2/3 activation is essential for the transdifferentiation of OGC‐deficient RPE cells.

Given the well‐established role of the TGF‐β/Smad pathway in EMT, we examined how this canonical pathway interacts with non‐canonical signaling in mediating EMT triggered by OGC deficiency in RPE cells. We observed a dose‐dependent increase in p‐p85, p‐AKT, p‐mTOR, and p‐Smad2/3 with higher PS concentrations, indicating coordinated activation (Figure 6E,F). Based on the crosstalk between these pathways, we hypothesized a summatory interaction between Smad2/3 and PI3K/AKT/mTOR signaling in mediating OGC deficiency‐induced EMT. To test this, OGC‐silenced RPE cells were treated with the PI3K inhibitor LY294002 (10 μM). OGC depletion caused a significant increase in p‐PI3K and p‐AKT/AKT ratios (*p* < 0.01, *p* < 0.01). LY294002 treatment effectively inhibited PI3K and AKT activation (Figure 6G,H). Moreover, PI3K inhibition significantly reduced the upregulation of p‐Smad2/3 and fibrotic markers (α‐SMA, Fibronectin, Collagen‐I), as well as the increased cell migration and proliferation associated with OGC deficiency (Figure S4A–D). To validate the in vitro findings, we measured both mitochondrial and total GSH levels in WT and OGC^+/−^ mice, before and after laser irradiation (Day 35 post laser). At baseline, OGC^+/−^ mice showed 67% lower mtGSH levels compared to WT mice (*p* < 0.01) (Figure 6I), while total GSH levels showed no difference between genotypes (Figure S5A). At 35 days post‐laser treatment, OGC^+/−^ mice exhibited significantly more pronounced GSH depletion, with mtGSH levels decreasing by 85.3% versus only 12.2% in WT mice (*p* < 0.01) (Figure 6I), and total GSH showing a 45.3% reduction in OGC^+/−^ mice compared to 32% in WT mice (*p* < 0.01) (Figure S5A), demonstrating that OGC deficiency exacerbates GSH depletion after retinal injury, particularly in the mitochondrial pool. In the same laser‐induced injury model, we detected activation of key signaling pathways consistent with our in vitro observations, including increased levels of TGF‐β2, p‐AKT, p‐Smad2/3, and p85. These markers were even more strongly upregulated in OGC^+/−^ mice 35 days after laser injury (Figure 6J,K). In parallel, we observed significantly larger lesions, increased collagen accumulation (Figure S5C–E), marked upregulation of the fibrotic marker α‐SMA, and a reduction in the epithelial marker E‐cadherin (Figure S5F) in OGC^+/−^ mice. Collectively, these findings suggest that Smad2/3 and PI3K signaling play a crucial role in mediating OGC‐deficiency‐induced EMT, likely acting in concert with the canonical TGFβ2/Smad pathway.

## 4 Discussion

This study presents novel data demonstrating the functional role of the anion transporter OGC in RPE cell EMT and its association with subretinal fibrosis. We show that OGC silencing exacerbates EMT, while overexpression attenuates TGF‐β2‐induced RPE EMT. Our results further reveal that OGC depletion significantly increases mtROS, reduces mtGSH, and mitochondrial bioenergetics, potentially contributing to RPE EMT. Mechanistically, OGC depletion increases TGF‐β2, promoting RPE cell proliferation and migration through Slug‐mediated EMT. Furthermore, we show that OGC depletion stimulates EMT via pSmad 2/3 upregulation, a process dependent on the PI3K/AKT signaling pathway. Importantly, OGC overexpression mitigates these effects. In vivo studies further demonstrate that subretinal fibrosis was significantly augmented in OGC^+/−^ mice via TGF‐β2‐dependent PI3K signaling.

Although OGC^+/−^ mice do not exhibit obvious retinal abnormalities or fibrosis under baseline conditions (Figure 1, Figure S1), this does not necessarily indicate the absence of underlying cellular stress or vulnerability. Subtle dysfunctions may be present but effectively compensated for, at least temporarily, by adaptive responses. To determine whether any retinal or RPE structural or functional impairments exist, more detailed ultrastructural examinations and functional assays will be essential. OGC serves a dual function, facilitating both α‐ketoglutarate transport and mitochondrial glutathione import. Disruption of either role can interfere with mitochondrial metabolism and redox regulation, two processes that are closely interdependent. The mitochondrial dysfunction observed in OGC^+/−^ mice likely results from a combined impact: diminished energy substrate availability and compromised antioxidant defenses (Mari et al. 2013; Ribas et al. 2014). Nevertheless, additional confirmatory studies are necessary to fully substantiate this hypothesis.

Our investigations exploring the natural progression of nAMD demonstrate a characteristic progression from CNV to a terminal stage marked by fibrous tissue formation. OGC^+/−^ mice showed a CNV profile like WT mice, with a peak at Day 7 post‐laser followed by CNV regression. However, fibrous tissue formation continued to increase significantly in OGC^+/−^mice from Day 21 and persisted until the end of the experiment on Day 35. In contrast, WT mice showed a peak in fibrous tissue formation by 21, followed by a steady state until the end of the experiment (Day 35), as previously reported (Ishikawa, Sreekumar, et al. 2016). The persistent fibrosis observed in OGC^+/−^ mice aligns with previous reports of significantly lower GSH levels in idiopathic pulmonary fibrosis (Beeh et al. 2002), and liver fibrosis (Fraser et al. 2022; Huang et al. 2023), suggesting a potential role for GSH in this process. The non‐regressing and potentially progressive nature of the lesions in OGC^+/−^ mice may have relevance to the chronic pathology observed in subretinal fibrosis and persistent fibrovascular membranes (SFB) during aging. The sustained mitochondrial redox imbalance, impaired glutathione transport, and increased TGF‐β2 levels in OGC^+/−^ mice could contribute to a persistent pro‐fibrotic and inflammatory microenvironment, which may hinder lesion resolution and promote fibrotic remodeling. This phenotype is reminiscent of the subretinal fibrotic changes and SFB observed in aged individuals and in models of chronic retinal injury, where ongoing oxidative stress and inflammation have been implicated in fibrosis (Ishikawa, Kannan, et al. 2016; Little et al. 2020; Wang et al. 2025; Zhang et al. 2025; Zhao et al. 2011). Thus, the non‐regressing lesion phenotype in OGC^+/−^ mice may reflect a TGF‐β2 driven chronic, redox fibrotic response relevant to human retinal disease.

Due to the complex nature of subretinal fibrosis pathogenesis, the underlying mechanism remains unclear. Myofibroblasts are the major pathogenic cells driving subretinal fibrosis by proliferating and synthesizing excess extracellular matrix proteins. However, the origin of myofibroblast within the subretinal fibrosis remains poorly defined. Previous studies have demonstrated that RPE cells, present in epiretinal and subretinal membranes, can lose their differentiated phenotype and transform into mesenchymal‐like cells, which are thought to initiate fibrosis (Hirasawa et al. 2011; Lopez et al. 1996). In this study, the effect of OGC suppression on the RPE EMT process may contribute to severe fibrosis formation. Cultured RPE cells transformed from typical epithelial cell morphology to a mesenchymal phenotype, characterized by spindled or stellate shapes, when OGC was silenced. These changes were more prominent under TGF‐β2 cotreatment. These results confirm that the OGC carrier plays an important role in RPE EMT.

OGC catalyzes the transport of 2‐oxoglutarate across the inner mitochondrial membrane in an electroneutral exchange for a dicarboxylate (e.g., malate) and plays an important role in several metabolic processes (Gutierrez‐Aguilar and Baines 2013; Odegaard et al. 2010; Yuan et al. 2022). OGC has been reported in porphyrin transport (Kabe et al. 2006), regulation of apoptosis, modulation of mitochondrial morphology (Gallo et al. 2011), and ferroptosis (Jang et al. 2021; Ta et al. 2022). We and others have confirmed that OGC functions as a critical mitochondrial GSH transporter in multiple tissues and cell lines (Chen and Lash 1998; Putt et al. 2012; Sreekumar et al. 2020; Ta et al. 2022; Wang et al. 2019; Wilkins et al. 2013; Zhong et al. 2008). Furthermore, our group has demonstrated the role of OGC in regulating mitochondrial metabolism and RPE monolayer health via mitochondrial bioenergetics, biogenesis, and barrier function.

It is well known that mitochondrial dysfunction is involved in nAMD (Brown et al. 2019; Kaarniranta et al. 2020), but the link between mitochondrial function and EMT in RPE cells remains unclear. Recent studies reveal the role of mitochondrial metabolic plasticity in the EMT process of RPE (Homma et al. 2021; Ma et al. 2024; Shu et al. 2021). Our study demonstrated a mechanism by which OGC deficiency contributes to RPE EMT and associated fibrotic changes. Silencing OGC and co‐treating with TGF‐β2 impaired mitochondrial function, as shown by increased mtROS and decreased MMP, consistent with our previous findings of reduced mitochondrial bioenergetics (Sreekumar et al. 2020). Additionally, similar mitochondrial dysfunction and a metabolic shift toward glycolysis during EMT have been reported (Ma et al. 2024; Shu et al. 2021). OGC inhibition led to mtGSH depletion, potentially disrupting redox homeostasis and promoting EMT progression, as supported by reports of decreased GSH in human and experimental fibrosis models (Liu and Gaston Pravia 2010). Notably, TGF‐β2 treatment further decreased the mtGSH in OGC silenced cells, impairing mitochondrial redox balance. Previous studies showed no changes in mitochondrial DNA copy number following OGC inhibition (Wang et al. 2019), suggesting mitochondrial dysfunction, rather than damage to the mitochondrial genome, caused the decline in respiratory function. These findings are corroborated by studies in cardiomyocytes (Jang et al. 2021) and rat brains (Miniero et al. 2021), where OGC inhibition increased mtROS, depolarized membranes, depleted GSH, and disrupted the malate/aspartate shuttle. Overall, OGC loss triggers mitochondrial dysfunction and metabolic reprogramming, and OGC overexpression reverses these effects, inhibiting EMT, reducing mtROS, restoring mtGSH, and rescuing mitochondrial function. However, further study is needed to fully understand the complex mechanisms of metabolic reprogramming in TGF‐β2‐treated cells and the precise role of OGC in this process.

EMT plays a critical role in the pathological progression of subretinal fibrosis in nAMD. Previous studies have shown that EMT is regulated by key transcriptional factors such as Snail, Zeb, and Twist (Fazilaty et al. 2019; Hirasawa et al. 2011; Ishikawa, Sreekumar, et al. 2016). Our findings align with these, showing significant activation of Slug upon OGC inhibition, accompanied by E‐cadherin down‐regulation, a hallmark of Slug‐mediated EMT (Palma‐Nicolas and Lopez‐Colome 2013). In vivo, we observed increased Slug expression (but not Snail) in OGC^+/−^ mice. Slug inhibition effectively reduced cell proliferation, migration, and EMT, suggesting Slug upregulation is a key mediator in subretinal fibrosis pathogenesis.

EMT can be activated by multiple signaling pathways, with the Smad2/3 pathway playing a key role through crosstalk with other signaling pathways (Guo and Wang 2009). In the present study, we observed increased phosphorylation of Smad2/3 in OGC‐silenced and PS‐treated RPE cells, indicating that OGC suppression induces RPE EMT via the Smad 2/3 pathway, a key event in canonical TGF‐β signaling (Akhurst and Hata 2012; Zhou et al. 2020). This suggests that altered redox homeostasis due to OGC interacts with TGF‐β/Smad 2/3 signaling during EMT. TGF‐β elevates ROS production by increasing NADPH oxidase 4 or inhibiting GSH biosynthesis, while ROS can, in turn, induce or activate TGF‐β from its latent state (Cao et al. 2022; Kim et al. 2020). Future research will explore the role of Smad4, which has been implicated in hRPE EMT (Wada et al. 2022).

The PI3K/Akt pathway is a critical mediator of TGF‐β‐induced EMT responses (Zhang et al. 2018; Zhao et al. 2022) with inhibition of PI3K/Akt blocking TGF‐β‐induced transcription, EMT, and cell migration (Han et al. 2023). Crosstalk between TGF‐β/Smad proteins and PI3K pathways has been documented in various cell types, including fibroblasts, keratinocytes, hepatic stellate cells, and cancer cells (Guo and Wang 2009). Both in vivo and in vitro studies have shown that PI3K/Akt inhibition was effective for controlling CNV in nAMD (Husain et al. 2022). Recent reports also suggest that aberrant Akt2 signaling in RPE potentially contributes to retinal fibrosis in diabetic retinopathy (Daley et al. 2023). Based on these studies, we confirmed that PI3K/Akt/mTOR signaling is involved in EMT induced by OGC depletion. Phosphorylation of Smad2/3 and Akt exhibits a dose‐dependent response to OGC inhibition, and knocking down of Smad2/3 or inhibiting PI3K/Akt reduced EMT in OGC‐deficient RPE cells. Higher concentrations of PS increased phosphorylation of ERK1/2 and GSK3β. The Akt/mTOR pathway is implicated in TGF‐β‐promoted pericyte‐myofibroblast transition subretinal fibrosis (Zhao et al. 2022), and suppressed GSK3β expression and activated PI3K/Akt signaling have been observed in proliferative vitreoretinopathy models (Zhang et al. 2018). Further research will investigate the role of PI3K/Akt signaling in EMT regulation by OGC.

Given its central role in maintaining mitochondrial integrity, OGC is an attractive therapeutic target for retinal and other fibrotic diseases associated with chronic oxidative stress. When OGC is inhibited or deficient, TGF‐β2 levels increase, mitochondrial GSH levels fall, leading to oxidative stress, mitochondrial dysfunction, and the activation of redox‐sensitive signaling pathways that promote EMT and fibrosis. Augmentation of the mitochondrial GSH pool through upregulation or activation of GSH transporters could provide a valuable approach to preventing retinal diseases linked to mitochondrial dysfunction such as fibrosis. Long‐term, restoring or enhancing OGC activity through gene therapy, mRNA delivery, or small‐molecule activation represents a more targeted and disease‐modifying strategy in fibrotic conditions where OGC loss contributes to pathogenesis.

While this study provides novel insights into the role of OGC in RPE EMT and subretinal fibrosis, it has limitations. OGC is also expressed in other retinal and choroidal cells that may also contribute to subretinal fibrosis progression, but these cell types were not addressed here. Although we did not observe any obvious retinal phenotype using standard methods, we agree that subtle RPE abnormalities might have gone undetected. To address this, our future studies will use higher‐resolution techniques like transmission electron microscopy (TEM) to examine ultrastructural changes, as well as ERG to assess retinal and RPE function more thoroughly. Additionally, the role of OGC in angiogenesis, particularly at earlier time points such as Days 7 and 14 post‐laser, is not yet understood and represents an exciting area for further investigation. While cell densities used in the study were selected based on assay‐specific requirements, differences in cell density may influence outcomes by affecting paracrine signaling, metabolic activity, and other cellular processes. Future studies could systematically evaluate the impact of cell density on the functional role of the anion transporter OGC in RPE cell EMT and its association with subretinal fibrosis. Additionally, although other SLC25 family members, like SLC25A39 (Liu, Liu, et al. 2023; Wang et al. 2021) and SLC25A10 (Chen and Lash 1998; Wang et al. 2019; Wilkins et al. 2013), are involved in mitochondrial GSH transport, their roles in the absence of OGC were not investigated. The present study aimed to elucidate the specific role of OGC in EMT and subretinal fibrosis. Moreover, while mtGSH is crucial, other mitochondrial antioxidants such as MnSOD, thioredoxins, glutaredoxins and catalase were not comprehensively examined.

In conclusion, this study highlights OGC as a key regulator of RPE EMT and subretinal fibrosis development. Loss of OGC exacerbates subretinal fibrosis in vivo and promotes RPE EMT through upregulation of Smad2/3 and activation of PI3K/Akt signaling pathways. Mitochondrial oxidative stress and dysfunction in OGC‐deficient cells suggest a mechanistic link to EMT. Notably, OGC overexpression reduces TGF‐β2‐induced proliferation, migration, and EMT, suggesting OGC as a potential therapeutic target for managing subretinal fibrosis in nAMD patients.

## Author Contributions

Mo Wang performed the experiments and wrote the manuscript. Feng‐Juan Gao, Fangyuan Hu, and Jun Ma performed the experiments. Gezhi Xu, Boya Lei, and Wenyi Tang analyzed the data. Ram Kannan designed the study, contributed to the discussion, and edited the manuscript. S.R.S. reviewed and edited the manuscript. Parameswaran G. Sreekumar and Gezhi Xu designed the study, analyzed the data, and edited the manuscript. All authors had the opportunity to discuss the results and provide comments on the manuscript.

## Conflicts of Interest

Dr. Sadda reports receiving honoraria for serving as a consultant for Nanoscope, Allergan/Abbvie, Roche/Genentech, Novartis, Bayer, Regeneron, 4DMT, Oxurion, Amgen, Alnylam, Alexion, Apellis, Astellas/Iveric, NGMBio, Biogen, Boerhinger Ingelheim, Optos, iCare, Heidelberg, Topcon, Notal Vision, SpliceBio, Saligoen, ONL Therapeutics. All other authors declare no conflicts of interest.

## Supporting information


**Figure S1:** Genotyping and characterization of OGC deficient mice (OGC^+/−^). (A) Genotyping of OGC^+/−^ mice. (B) Protein expression of OGC in WT and OGC^+/−^ mice. Western blot analysis (B) indicated that protein expression levels of OGC were significantly decreased in the RPE‐choroid complexes of the OGC^+/−^ mice, with ~50% reduction in OGC protein levels compared to WT mice. (C) SD‐OCT horizontal B‐scan. Scale bar: 200 μm. (D–F) Choroidal capillary length and thickness measured by imageJ. No major changes in OCT or choroidal capillary length or thickness were observed in the OGC^+/−^ mice. (G) RT‐PCR analysis indicated that the mRNA expression levels of OGC were significantly decreased in the RPE‐choroid complexes of the OGC^+/−^ mice, with ~50% reduction in OGC mRNA compared to WT mice. (H) No significant changes in total GSH levels were observed between the WT and OGC^+/−^ mice at baseline. Data are presented as mean ± SD (*n* = 3), Student's *t*‐test, NS, not significant; **p < 0.01. (I) No primary control for Collagen I staining in RPE‐Choroid flat mount. Scale bar: 100 μm.
**Figure S2:** OGC inhibition aggravated EMT, whereas overexpression attenuated TGF‐β2‐induced EMT. (A) ARPE‐19 cells were treated with varying doses of PS (0, 2, 5, 10 mM) for 48 h. Samples were analyzed for EMT markers via qRT‐PCR for mRNA expression of E‐cadherin, α‐SMA, Collagen I, and Fibronectin. GAPDH served as the internal control. (B, C) OGC silenced or over expressed (OGC+), ARPE‐19 cells were stimulated with TGF‐β2 (10 ng/mL) for 48 h. The mRNA expression of E‐cadherin, α‐SMA and OGC with or without TGF‐β2 treatment normalized to GAPDH is shown. Data are presented as means± SD. *n* = 3 per group. NS, nonsignificant difference; *p < 0.05, **p < 0.01, ***p < 0.001.
**Figure S3:** OGC silencing aggravated EMT, whereas overexpression attenuated TGF‐β2‐induced EMT. (A) ARPE‐19 cells were stimulated with TGF‐β2 (10 ng/mL) for 48 h. Western blot analysis of OGC protein expression. (B) Quantification of OGC protein expression using ImageJ. Data are presented as mean ± SD. *n* = 3 per group. Student's *t*‐test, **p < 0.01. (C) ARPE‐19 cells with OGC silencing or overexpression (OGC+) were stimulated with TGF‐β2 (10 ng/mL) for 48 h and immunostained for E‐cadherin (red) and α‐SMA (green). Scale bar: 100 μm. (D) OGC silencing induced EMT‐associated morphological changes in ARPE‐19 cells, and TGF‐β2 treatment further enhanced these mesenchymal phenotypic changes. The majority of TGF‐β2‐treated and OGC‐silenced cells exhibited a spindle‐shaped, fibroblast‐like, mesenchymal phenotype. Images were acquired by phase‐contrast microscopy (20× magnification) from ≥ 3 independent experiments. Scale bar: 100 μm.
**Figure S4:** OGC depletion promotes EMT via upregulation of pSmad2/3‐dependent PI3K/AKT signaling pathway activation. ARPE‐19 cells were transfected with control siRNA or OGC siRNA for 48 h, followed by treatment with vehicle alone or LY294002 (10 μM, an inhibitor of phosphatidylinositol 3‐kinase) for 1 h. LY294002 significantly inhibited the activation of PI3 Kinase p85 and AKT in OGC deficient cells. (A) Samples were analyzed for EMT markers via qRT‐PCR for mRNA expression of E‐cadherin, α‐SMA, Collagen I, Fibronectin and pSmad2/3. GAPDH served as the internal control. PI3K inhibition significantly reduced the upregulation of p‐Smad2/3 and fibrotic markers (α‐SMA, Fibronectin, Collagen‐I). (B, C) Cell proliferation and (D) migration were significantly reduced with PI3K inhibition in OGC deficient cells (20× magnification, Scale bar: 100 μm). Data shown are mean ± SD, *n* = 3, NS, not significant; **p < 0.01, ***p < 0.001, ****p < 0.0001.
**Figure S5:** Subretinal fibrosis was significantly augmented in OGC^+/−^ mice following laser photocoagulation. (A) Total GSH levels in the RPE/choroid complex of WT and OGC^+/−^ mice before and 35 days after laser treatment. (B) RT‐PCR analysis of TGF‐β2 mRNA expression in WT and OGC^+/−^ mice before and 35 days after laser treatment. (C) Hematoxylin–eosin (H&E) (scale bar: 200 μm) and (D) Masson trichrome staining (scale bar: 100 μm) showing lesions in WT and OGC^+/−^ mice on Day 35 post‐laser. Yellow dotted lines and yellow asterisks indicate lesions. Red arrows indicating collagen (blue)‐rich lesions. (E) Quantification of fibrosis areas in Masson trichrome stained sections was performed using ImageJ (*n* = 8 mice per group); mean ± SEM, Unpaired *t*‐test, ***p < 0.001. (F) Representative confocal images (*n* = 3 retinal sections) of retinal cryosections from WT and OGC^+/−^ mice showed subretinal fibrotic lesions stained for E‐cadherin (green), α‐SMA (red) or no primary antibody. Cell nuclei were counterstained with DAPI (blue). Scale bar: 25 μm.

## Data Availability

The data that supports the findings of this study are available in the Supporting Information of this article.
