# Supplementary material for: Deficiency of 2‐Oxoglutarate Carrier (Slc25a11) Drives RPE Epithelial‐to‐Mesenchymal Transition and Exacerbates Subretinal Fibrosis in Neovascular Age‐Related Macular Degeneration
Source: Aging Cell. 2025 Oct 28;24(12):e70271. doi: 10.1111/acel.70271 (PMC12686559; doi:10.1111/acel.70271)
Supplement: Supplementary file 1 — Figure S1: Genotyping and characterization of OGC deficient mice (OGC+/−). (A) Genotyping of OGC+/− mice. (B) Protein expression of OGC in WT and OGC+/− mice. Western blot analysis (B) indicated that protein expression levels of OGC were significantly decreased in the RPE‐choroid complexes of the OGC+/− mice, with ~50% reduction in OGC protein levels compared to WT mice. (C) SD‐OCT horizontal B‐scan. Scale bar: 200 μm. (D–F) Choroidal capillary length and thickness measured by imageJ. No major changes in OCT or choroidal capillary length or thickness were observed in the OGC+/− mice. (G) RT‐PCR analysis indicated that the mRNA expression levels of OGC were significantly decreased in the RPE‐choroid complexes of the OGC+/− mice, with ~50% reduction in OGC mRNA compared to WT mice. (H) No significant changes in total GSH levels were observed between the WT and OGC+/− mice at baseline. Data are presented as mean ± SD (n = 3), Student's t‐test, NS, not significant; **p < 0.01. (I) No primary control for Collagen I staining in RPE‐Choroid flat mount. Scale bar: 100 μm. Figure S2: OGC inhibition aggravated EMT, whereas overexpression attenuated TGF‐β2‐induced EMT. (A) ARPE‐19 cells were treated with varying doses of PS (0, 2, 5, 10 mM) for 48 h. Samples were analyzed for EMT markers via qRT‐PCR for mRNA expression of E‐cadherin, α‐SMA, Collagen I, and Fibronectin. GAPDH served as the internal control. (B, C) OGC silenced or over expressed (OGC+), ARPE‐19 cells were stimulated with TGF‐β2 (10 ng/mL) for 48 h. The mRNA expression of E‐cadherin, α‐SMA and OGC with or without TGF‐β2 treatment normalized to GAPDH is shown. Data are presented as means± SD. n = 3 per group. NS, nonsignificant difference; *p < 0.05, **p < 0.01, ***p < 0.001. Figure S3: OGC silencing aggravated EMT, whereas overexpression attenuated TGF‐β2‐induced EMT. (A) ARPE‐19 cells were stimulated with TGF‐β2 (10 ng/mL) for 48 h. Western blot analysis of OGC protein expression. (B) Quantification of OGC prote [file ACEL-24-e70271-s001.docx]

**Figure S1**


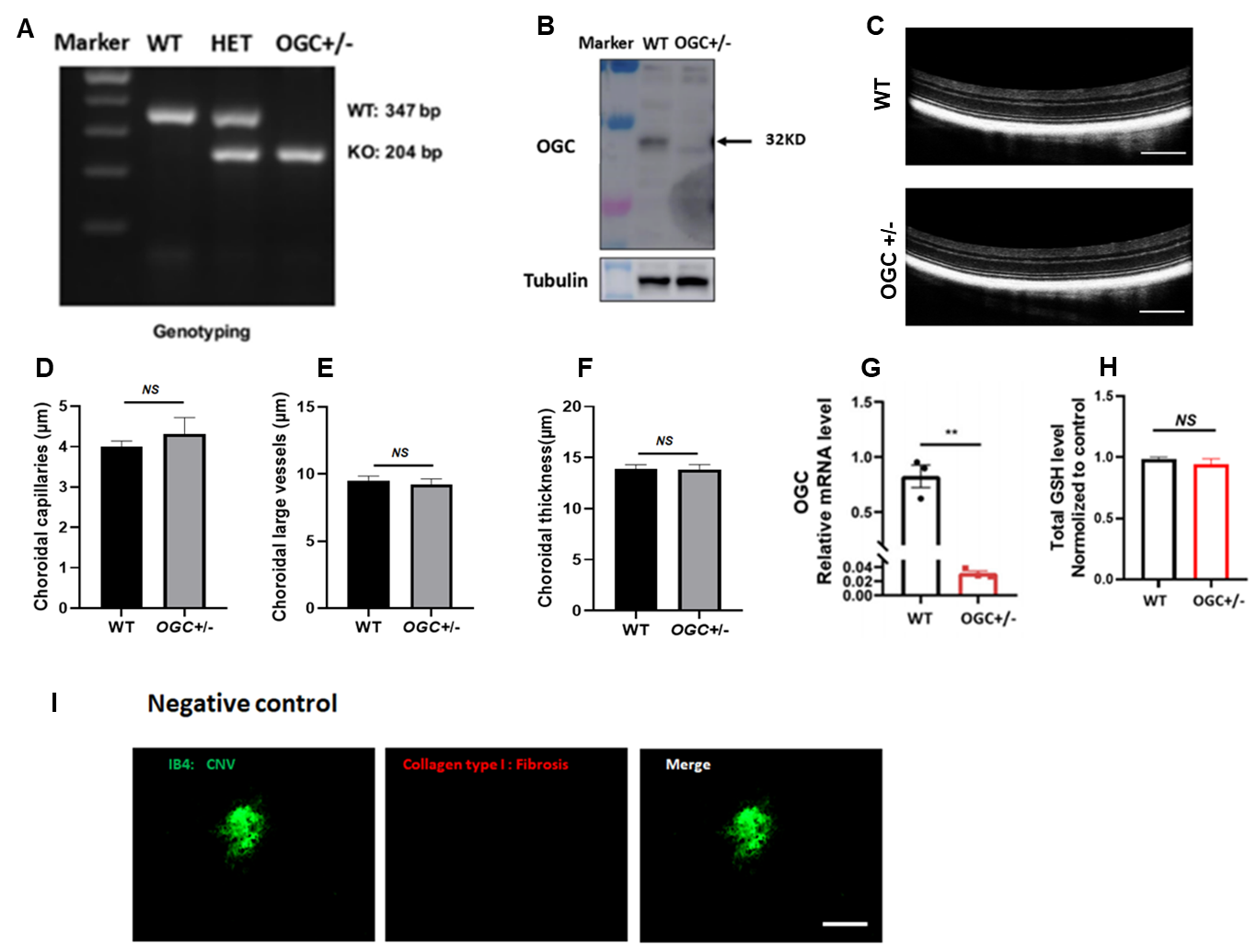


**Figure S1. Genotyping and characterization of OGC deficient mice (OGC^+/-^). (A)** Genotyping of OGC^+/-^ mice. **(B)** Protein expression of OGC in WT and OGC^+/-^ mice. Western blot analysis **(B)** indicated that protein expression levels of OGC were significantly decreased in the RPE-choroid complexes of the OGC^+/-^ mice, with ~50% reduction in OGC protein levels compared to WT mice. **(C)** SD-OCT horizontal B-scan. Scale bar: 200μm. (**D, E, F**) Choroidal capillary length and thickness measured by image J. No major changes in OCT or choroidal capillary length or thickness were observed in the OGC^+/-^ mice. **(G)** RT-PCR analysis indicated that the mRNA expression levels of OGC were significantly decreased in the RPE-choroid complexes of the OGC^+/-^ mice, with ~50% reduction in OGC mRNA compared to WT mice. (**H**) No significant changes in total GSH levels were observed between the WT and OGC^+/-^ mice at baseline. Data are presented as mean ± SD (n = 3), Student’s t-test, NS, not significant, **P < 0.01. (**I**) No primary control for Collagen I staining in RPE-Choroid flat mount. Scale bar: 100 μm.

**Figure S2**


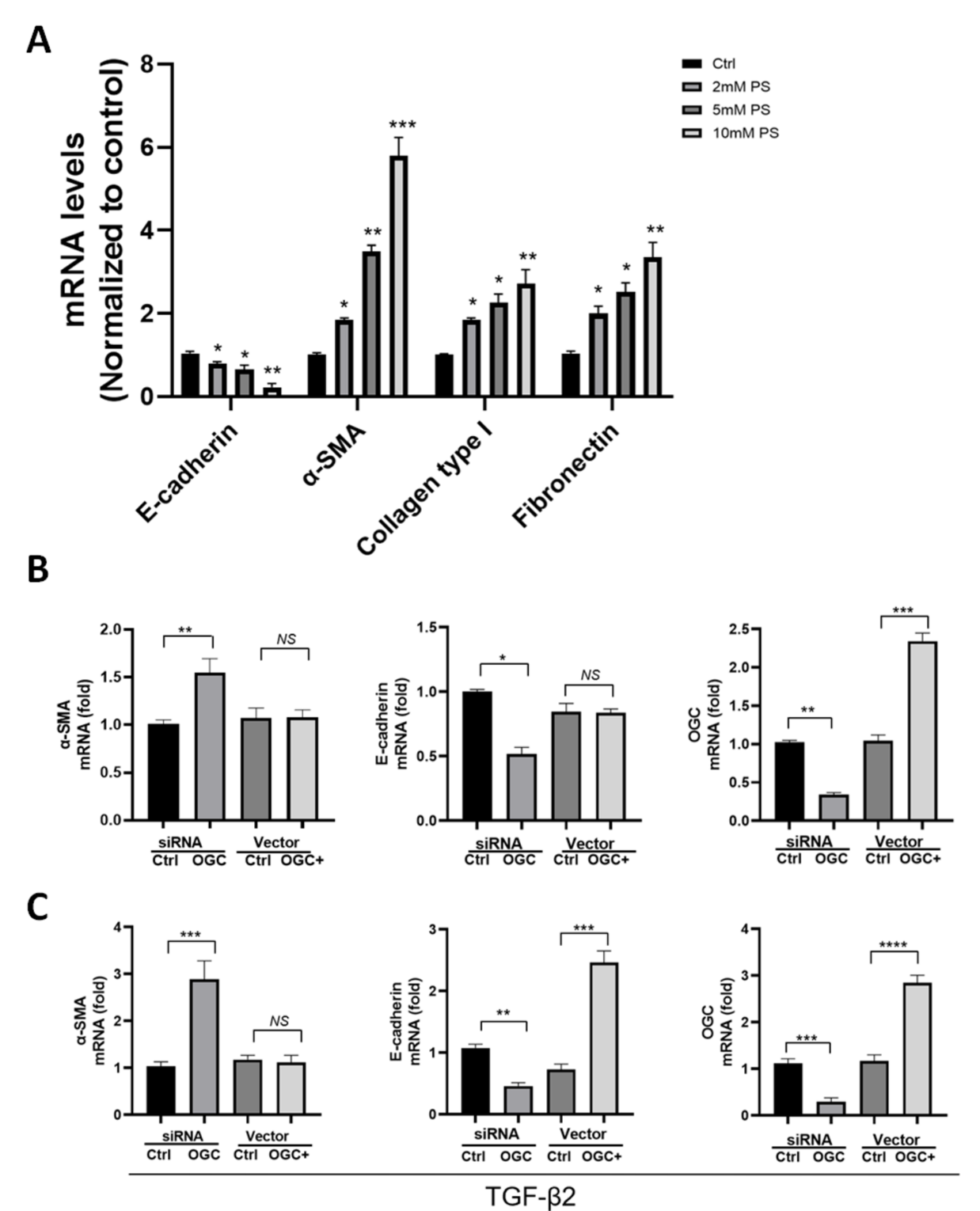


**Figure S2.** **OGC inhibition aggravated EMT, whereas overexpression attenuated TGF-β2-induced EMT.** (**A**) ARPE-19 cells were treated with varying doses of PS (0, 2, 5, 10 mM) for 48 hours. Samples were analyzed for EMT markers via qRT-PCR for mRNA expression of E-cadherin, α-SMA, Collagen I, and Fibronectin. GAPDH served as the internal control. (**B, C**) OGC silenced or over expressed (OGC+), ARPE-19 cells were stimulated with TGF-β2 (10 ng/mL) for 48 hours. The mRNA expression of E-cadherin, α-SMA and OGC with or without TGF-β2 treatment normalized to GAPDH is shown. Data are presented as means± SD. n = 3 per group. *NS*, nonsignificant difference, *P < 0.05, **P < 0.01, ***P < 0.001.

**Figure S3**


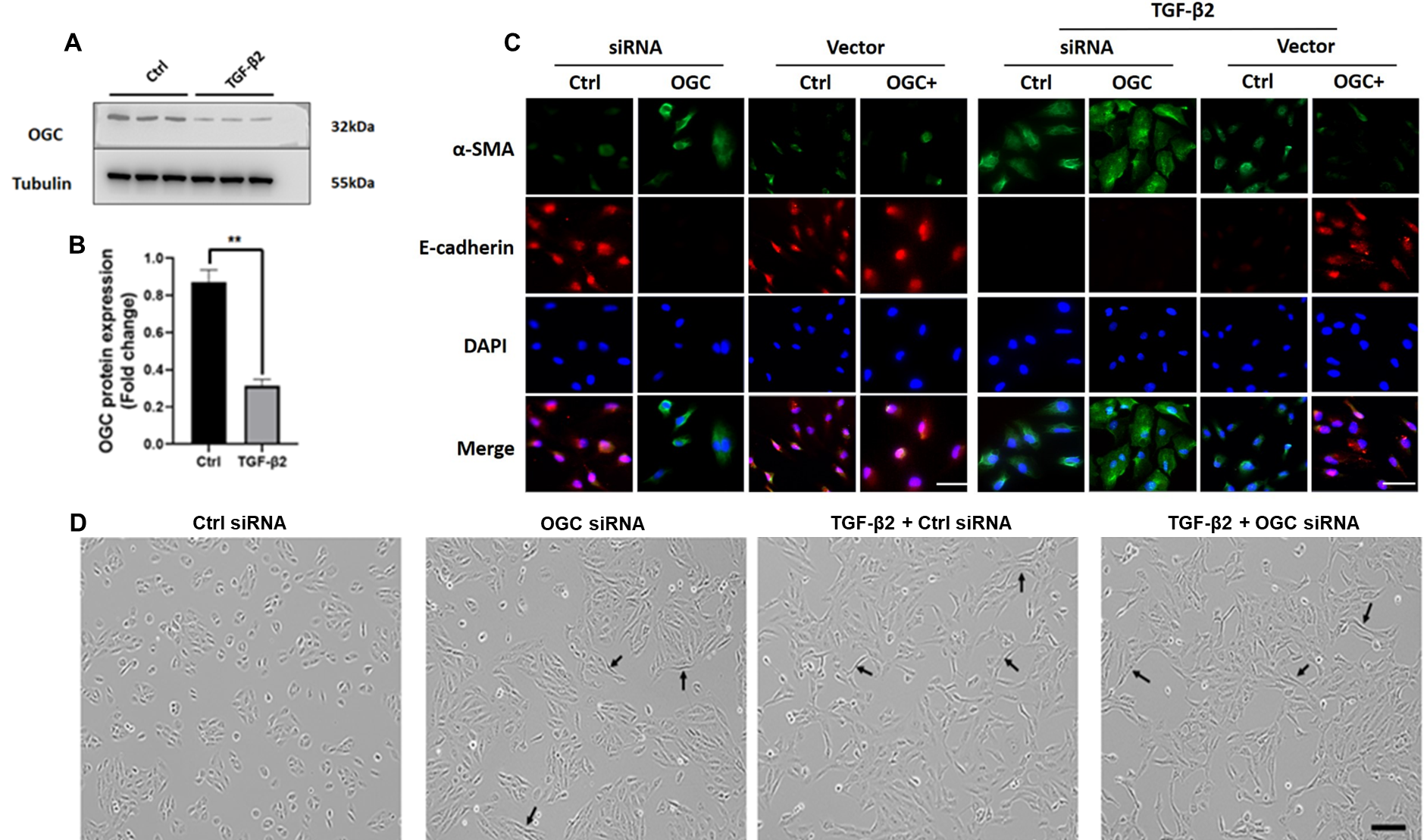


**Figure S3. OGC silencing aggravated EMT, whereas overexpression attenuated TGF-β2-induced EMT.** (**A**) ARPE-19 cells were stimulated with TGF-β2 (10 ng/mL) for 48 hours. Western blot analysis of OGC protein expression. (**B**) Quantification of OGC protein expression using ImageJ. Data are presented as mean ± SD. n = 3 per group. Student’s *t*-test, **p < 0.01. (**C**) ARPE-19 cells with OGC silencing or overexpression (OGC+) were stimulated with TGF-β2 (10 ng/mL) for 48 hours and immunostained for E-cadherin (red) and α-SMA (green). Scale bar: 100 μm. (**D**) OGC silencing induced EMT-associated morphological changes in ARPE-19 cells, and TGF-β2 treatment further enhanced these mesenchymal phenotypic changes. The majority of TGF-β2-treated and OGC-silenced cells exhibited a spindle-shaped, fibroblast-like, mesenchymal phenotype. Images were acquired by phase-contrast microscopy (20× magnification) from ≥3 independent experiments. Scale bar: 100 μm.

**Figure S4**


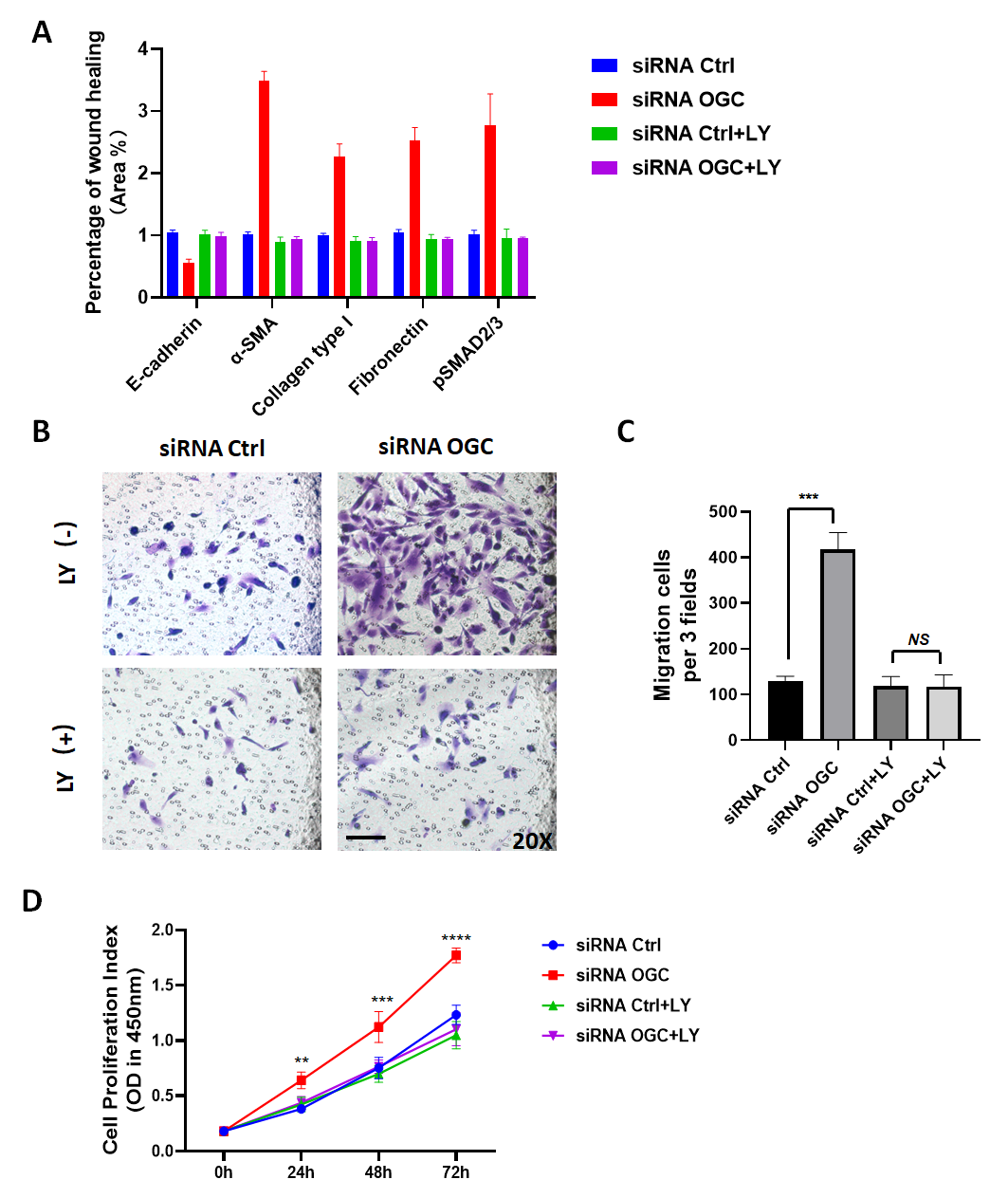


**Figure S4. OGC depletion promotes EMT via upregulation of pSmad2/3-dependent PI3K/AKT signaling pathway activation.** ARPE-19 cells were transfected with control siRNA or OGC siRNA for 48 hours, followed by treatment with vehicle alone or LY294002 (10 μM, an inhibitor of phosphatidylinositol 3-kinase) for 1 hour. LY294002 significantly inhibited the activation of PI3 Kinase p85 and AKT in OGC deficient cells. (**A**) Samples were analyzed for EMT markers via qRT-PCR for mRNA expression of E-cadherin, α-SMA, Collagen I, Fibronectin and pSmad2/3. GAPDH served as the internal control. PI3K inhibition significantly reduced the upregulation of p-Smad2/3 and fibrotic markers (α-SMA, Fibronectin, Collagen-I). (**B, C**) Cell proliferation and **(D)** migration were significantly reduced with PI3K inhibition in OGC deficient cells (20× magnification, Scale bar: 100 μm). Data shown are mean ± SD, n =3, NS, not significant, **P < 0.01, ***P < 0.001, ****P < 0.0001.

**Figure S5**


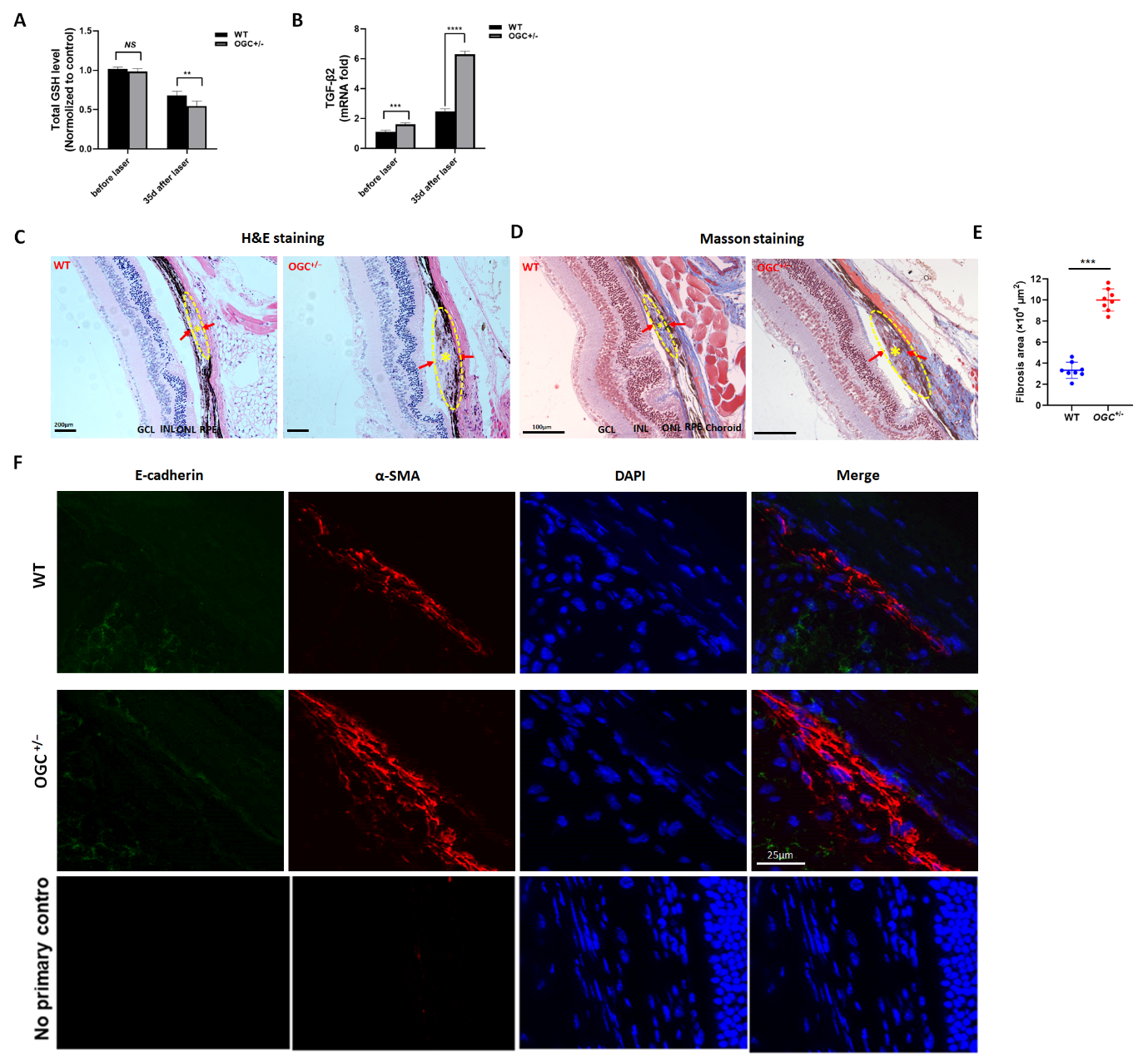


**Figure S5. Subretinal fibrosis was significantly augmented in OGC**^+/-^ **mice following laser photocoagulation**. (**A**) Total GSH levels in the RPE/choroid complex of WT and OGC^+/−^ mice before and 35 days after laser treatment. (**B**) RT-PCR analysis of TGF-β2 mRNA expression in WT and OGC^+/-^ mice before and 35 days after laser treatment. (**C**) Hematoxylin-eosin (H&E) (scale bar: 200 µm) and (**D**) Masson trichrome staining (scale bar: 100µm) showing lesions in WT and OGC^+/-^ mice on day 35 post-laser. Yellow dotted lines and yellow asterisks indicate lesions. Red arrows indicating collagen (blue)-rich lesions. (**E**) Quantification of fibrosis areas in Masson trichrome stained sections was performed using Image J (n = 8 mice per group; mean ± SEM, Unpaired t-test, ***P < 0.001. (**F**) Representative confocal images (n=3 retinal sections) of retinal cryosections from WT and OGC^+/-^ mice showed subretinal fibrotic lesions stained for E-cadherin (green), α-SMA (red) or no primary antibody. Cell nuclei were counterstained with DAPI (blue). Scale bar: 25 µm.
